# A multidimensional analysis of the 21^st^ century competencies scale through ai-driven data mining techniques

**DOI:** 10.1038/s41598-025-27568-8

**Published:** 2025-12-11

**Authors:** Nigmet Koklu

**Affiliations:** Vocational School of Technical Sciences, Konya Technical University, Konya, Türkiye Turkey

**Keywords:** 21^st^ century competencies, Classification, Educational assessment, Grid search optimization, Machine learning, Engineering, Mathematics and computing

## Abstract

In recent years, evaluating competencies such as knowledge, practical skills, character traits, and meta-learning capabilities has gained increasing importance in educational research. As educational datasets grow larger and more complex, machine learning offers promising tools for analyzing student responses and identifying patterns that support assessment processes. This study aims to classify student responses collected through the 21st Century Competencies Scale using a variety of machine learning algorithms, including SVM, ANN, k-NN, RF, LR, DT, AdaBoost, Gradient Boosting, and XGBoost. The dataset contains responses from 616 participants and covers four key sub-dimensions. Model performance was measured using accuracy, precision, recall, and F1-score. Grid search optimization was also applied to improve performance. The highest classification accuracy was achieved by LR in the “Character” sub-dimension (78.73%), followed by SVM in the “Skills” (78.58%) and overall scale (74.51%). Gradient Boosting and k-NN models also showed competitive results across multiple dimensions. These findings emphasize the effectiveness of machine learning, particularly when combined with parameter optimization, in supporting data-driven educational assessments.

## Introduction

Today’s education approach goes beyond simply imparting academic knowledge and aims to equip individuals with multifaceted competencies that can adapt to the complexities of the 21 st century^1^. These competencies include skills such as critical thinking, problem-solving, communication, collaboration, creativity, ethical values, and self-direction^2^. Equipping students with these skills ensures they are prepared for the rapid changes in life and the business world. 21 st century competencies are examined in three groups: ways of thinking, work tools, and global living skills. Information literacy, technology use, global citizenship, and career management are discussed within this framework^3^. For students to acquire these competencies, teachers must be equipped with digital skills, communication skills, and lifelong learning. However, moving away from traditional teaching, integrating technology into lessons, and using student-centered methods create various challenges^4^. Therefore, supporting teachers with continuous professional development and effectively integrating 21 st century competencies into programs is crucial^5^.

Prospective teachers are expected to be able to address socio-scientific issues that require consideration of multiple perspectives and values, and to possess the ability to effectively analyze and synthesize information from diverse sources. Competencies such as working in multidisciplinary teams and solving ambiguous problems through the effective use of information and communication technologies are gaining importance. Furthermore, teachers must be developed as individuals who possess the capacity to adapt to future societies, succeed, and sustain lifelong learning^5^.

The integration of these competencies necessitates a fundamental paradigm shift in pedagogical approaches. The shift from traditional teacher-centered methods to student-centered and inquiry-based learning models forms the basis of this transformation. Accordingly, teacher preparation programs should ensure that candidates both gain personal competence in these competencies and effectively transfer these skills to students^6^. The learning process should be structured as student-centered, collaborative, and integrated with the social context, supporting the effective acquisition of 21 st century skills. The importance of these skills is linked to the need for individuals to innovate, adapt, and solve complex problems^2^.

The 21 st century, with its information society and digital transformation, expects education systems to equip individuals not only with academic knowledge but also with multifaceted skills such as critical thinking, creativity, communication, collaboration, digital literacy, and cultural awareness. These competencies must be addressed holistically, encompassing cognitive, interpersonal, intrapersonal, and technical dimensions, for individuals to play an effective role in the information society. Geisinger categorizes these skills under headings such as critical thinking, problem solving, self-regulation, communication, and digital literacy^7^. González-Pérez and Ramírez-Montoya emphasize that 21 st century competencies should be evaluated within the context of Education 4.0, particularly emphasizing the importance of supporting them with complex thinking, a systems approach, digital competencies, and active learning strategies^8^. Larson and Miller, on the other hand, argue that these skills are not new but have become “newly important” in today’s conditions, advocating that these competencies should be integrated across all disciplines and taught in technology-supported learning environments^9^. Similarly, Chalkiadaki points out that there are conceptual ambiguities and practical shortcomings in the integration of these skills into educational policies, particularly at the primary school level^10^. Allen and Van der Velden emphasize that 21st century competencies, along with core academic knowledge and field-specific skills, form a complementary whole and that teachers’ digital pedagogical competencies should be strengthened accordingly^11^. In a study conducted in the Turkish context, Agaoglu and Demir demonstrated through a “Where are we?” activity with “Bilim ve Sanat Eğitim Merkezlerinin” (BILSEM) students that interdisciplinary, student-centered, and activity-based approaches support 4 C skills such as creative thinking, critical thinking, communication, and collaboration^12^. All these studies collectively demonstrate that the acquisition of 21 st century competencies is possible not only at the curriculum level but also through the restructuring of classroom practices, teacher training, and assessment and evaluation systems.

The concept of 21 st century competencies has been widely discussed in international educational frameworks. According to the OECD Learning Compass 2030, these competencies encompass knowledge, skills, attitudes, and values that prepare individuals to shape a sustainable and inclusive future^13^. UNESCO highlights *global competence* as the capacity to examine local, global, and intercultural issues, while the Partnership for 21 st Century Learning (P21) emphasizes the integration of core knowledge, critical thinking, communication, collaboration, and creativity. In line with these frameworks, the scale used in this study evaluates four major dimensions:

- **Knowledge**, referring to mastery of core disciplinary and interdisciplinary content.
- **Skills**, including problem-solving, critical thinking, communication, and collaboration abilities.
- **Character**, encompassing personal and ethical dispositions such as responsibility, resilience, and empathy.
- **Meta-learning**, representing the capacity for learning how to learn, adaptability, and self-regulation.

Although these international frameworks broadly converge on the importance of holistic 21 st -century competencies, there remain conceptual ambiguities and differences in emphasis across contexts. For example, OECD emphasizes adaptability and sustainability, while UNESCO highlights intercultural understanding, and P21 focuses more strongly on the “4Cs” (critical thinking, communication, collaboration, creativity)^14^. Such variations influence how competencies are interpreted, prioritized, and implemented in national educational policies, which indicates that the concept is still evolving and subject to ongoing debate^15^.

Beyond summary-score modeling typical of traditional psychometrics, AI-driven educational data mining can capture non-linear, cross-dimension interactions, leverage cross-validated prediction for out-of-sample performance, and surface individualized risk or support profiles. Such capabilities make AI a natural complement to reliability/validity-oriented psychometric analysis when the goal is accurate multi-class classification of competency levels and decision support for instructors and advisors^16^. Recent syntheses in learning analytics and psychometric machine learning research underscore this complementary role and report practical gains in classification and early-warning settings^17^.

Unlike traditional psychometric approaches, which primarily rely on summary scores and may fail to capture complex, multidimensional relationships within competency data, artificial intelligence based: methods provide the ability to detect nuanced patterns and interactions across multiple sub-dimensions^18^. This allows for more accurate classification of students’ competencies, supports predictive modeling, and enables insights that can guide targeted educational interventions^19^. Beyond traditional static feature selection techniques, dynamic and adaptive approaches have recently been proposed to enhance predictive performance in educational data mining^20^.

This theoretical and methodological grounding establishes a strong foundation for analyzing competency data with machine learning techniques, ensuring that the study’s findings are both aligned with international educational standards and enriched with actionable insights for educators and policymakers.

## Materials and methods

**Fig. 1:**
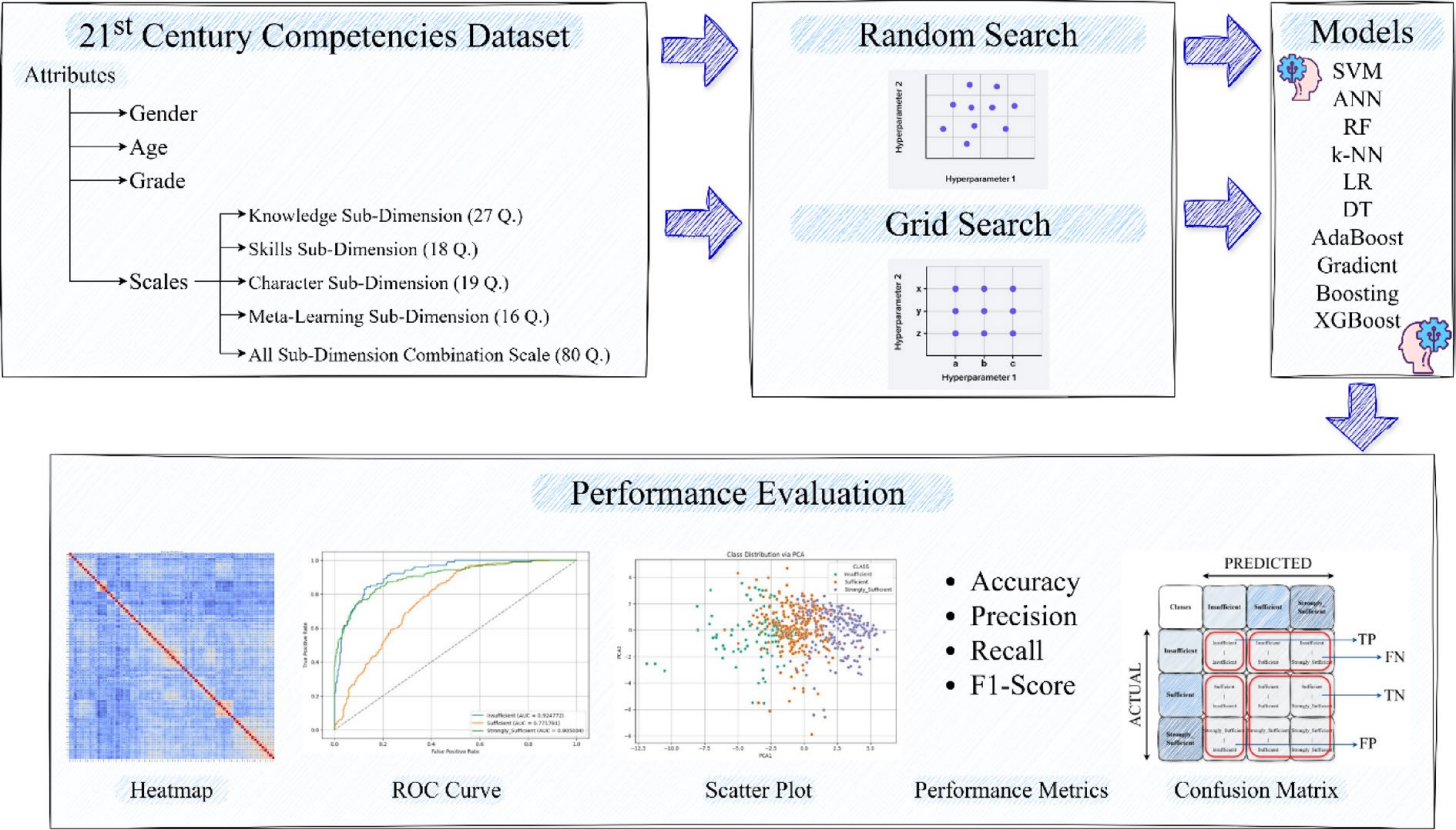
Flow diagram for 21 st century competencies scales study.

This section describes the 21 st Century Competencies Dataset, its configuration matrix, performance metrics, cross-validation, data mining methods, heatmap, and grid search. The dataset was generated using the 21 st Century Competencies scale. Performance metrics such as accuracy, F1 score, precision, and recall, as well as data mining methods such as Support Vector Machine (SVM), Artificial Neural Network (ANN), Random Forest (RF), k-Nearest Neighbor (k-NN), Logistic Regression (LR), Decision Tree (DT), AdaBoost, Gradient Boosting, and Extreme Gradient Boosting (XGBoost) are discussed in detail. The flow diagram for the article is shown in Fig. 1.

### Dataset for “21st century competencies (21st CC Dataset)”

This study examined numerical data related to the 21 st century competencies of university students. Classification was performed on the dataset prepared by us using various classification algorithms. The flow model illustrating the study’s process is presented in Fig. 1. Examining the structure of the figure, the first step requires obtaining the data. The obtained data was subjected to various data preprocessing steps, and 84 different features were extracted at the end of this process. In this study, we deliberately relied on self-reported survey data rather than behavioral traces, which provides standardized but declarative measures of competencies. These features were then transferred to the dataset. Then, the data was trained repeatedly using the cross-validation method and fed into the classification models. The trained algorithms produced outputs according to the specified classes.

The dataset used in the study was obtained using the 21 st century competencies scale developed by Yilmaz and Alkis^21^. The scale has demonstrated strong psychometric properties: Cronbach’s alpha coefficients ranged from 0.82 to 0.91 across the four sub-dimensions, confirming internal consistency reliability. Construct validity was also supported through exploratory and confirmatory factor analyses, justifying the use of the combined 80-item instrument in this study. The authors determined four sub-dimensions in the scale: knowledge, skills, character, and meta-learning. The knowledge sub-scale consists of 27 items, the skills sub-scale consists of 18 items, the character sub-scale consists of 19 items, and the meta-learning sub-scale consists of 16 items. The authors stated that the four sub-dimensions can also be used separately for different purposes. In this study, an 80-item scale with all sub-dimensions combined chosen to use, in line with our purpose. During the data collection process, first, three questions regarding demographic characteristics (gender, age, and grade) were asked, and then an 80-item scale was applied, ultimately creating a three-class dataset. The 21 st CC Dataset contains 84 variables that can determine individuals’ 21 st century competencies. The data was collected by the researcher from 616 university students studying at various universities in Turkey between April 10, 2025, and May 20, 2025. The attributes and values for the dataset are shown in Table 1. The demographic composition of the participants is presented in Table 1. The sample included both male and female students, aged between 18 and 28 years and represented all undergraduate grade levels (Year 1–Year 5).

Data preprocessing included several steps to ensure data quality. Initially, all responses were screened for missing values; incomplete submissions (*n* = 2) were removed rather than imputed, as their negligible proportion would have introduced artificial variance if imputed. Categorical variables such as gender and grade were label-encoded. All Likert-scale items (1–5) were deliberately retained in their original ordinal form without normalization, in order to preserve the inherent rank-order meaning of the categories. Class imbalance was minimal (101 Insufficient, 298 Sufficient, 217 Strongly Sufficient), so no re-sampling was required. Outlier detection was conducted using both z-score and IQR thresholds, but no responses exceeded the valid ranges. In total, the dataset contained 84 variables (80 items plus 4 demographic attributes), which were compiled for analysis.

To categorize students into three distinct competency levels (*Insufficient*,* Sufficient*,* Strongly Sufficient*), each participant’s mean score across the 80 Likert-type items was calculated (responses coded 1 = strongly disagree to 5 = strongly agree). Based on the frequency distribution of these mean scores, natural clusters were observed and used to define the thresholds:

- **Insufficient**: 2.60–3.74.
- **Sufficient**: 3.75–4.24.
- **Strongly Sufficient**: 4.25–4.88.

**Fig. 2:**
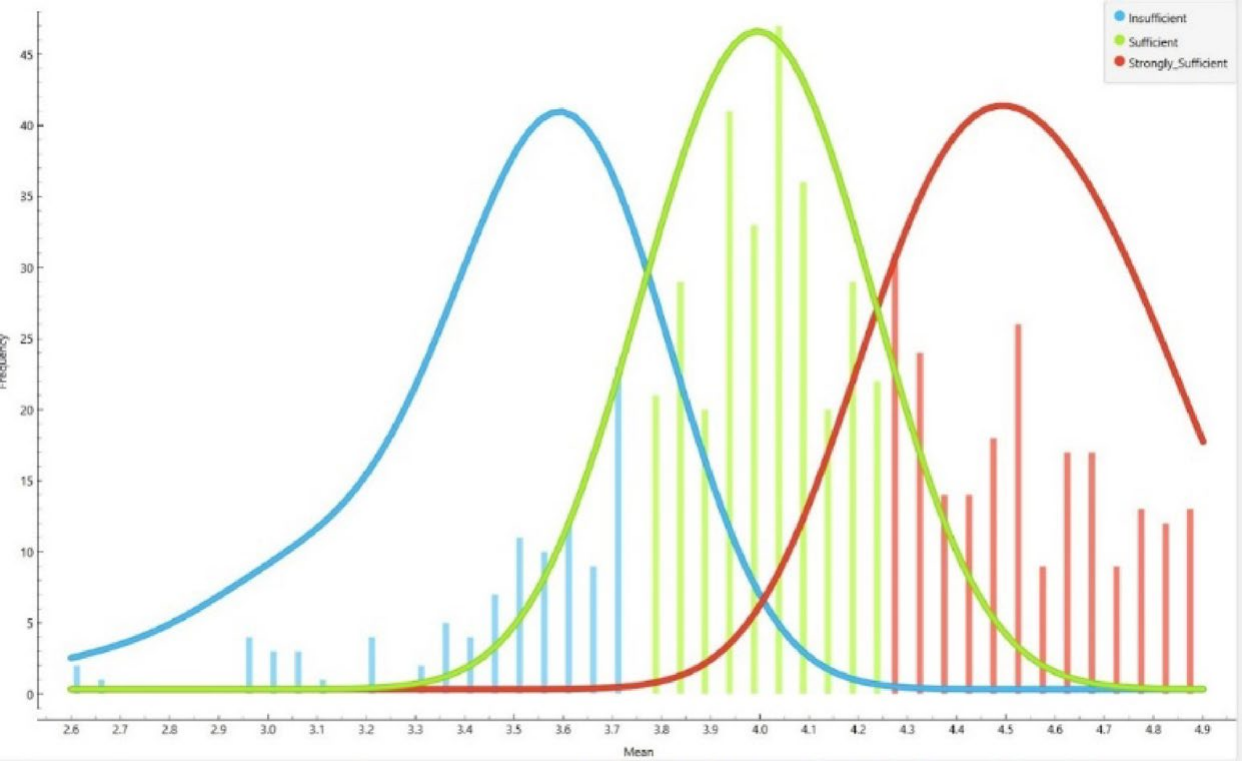
Frequency distribution of mean competency scores showing the three class thresholds.

This procedure reflects the ordinal nature of Likert-type data, avoids arbitrary cut-offs, and ensures reproducibility. It should be noted that the dataset is entirely based on self-reported Likert-type questionnaire responses. While this allows for structured and standardized measurement, it also limits the scope of inference compared to behavioral or digital trace data. This limitation is acknowledged and discussed further in the conclusion. The clustering of responses within these ranges is illustrated in Fig. 2.

**Table 1:** Attributes and values of 21 st CC Dataset.

Attributes	Values	*N*
Gender	Male	526
	Female	90
Age	19–24	466
	25–30	107
	31–40	32
	41+	11
Grade	1 st year	12
	2nd year	187
	3rd year	119
	4th year	188
	5th year (extended)	110
Sub dimension − 1 (27 item)	1-Strongly disagree; 2-Disagree; 3-Neutral; 4-Agree; 5-Strongly agree	-
Sub dimension − 2 (18 item)	1-Strongly disagree; 2-Disagree; 3-Neutral; 4-Agree; 5-Strongly agree	-
Sub dimension − 3 (19 item)	1-Strongly disagree; 2-Disagree; 3-Neutral; 4-Agree; 5-Strongly agree	-
Sub dimension − 4 (16 item)	1-Strongly disagree; 2-Disagree; 3-Neutral; 4-Agree; 5-Strongly agree	-
21 st _century_competency (Classes)	1- Insufficient (This person has insufficient 21 st century competency)	101
	2- Sufficient (This person has sufficient 21 st century competency)	298
	3- Strongly sufficient (This person has strongly sufficient 21 st century competency)	217

### Confusion matrix and performance metrics

**Fig. 3:**
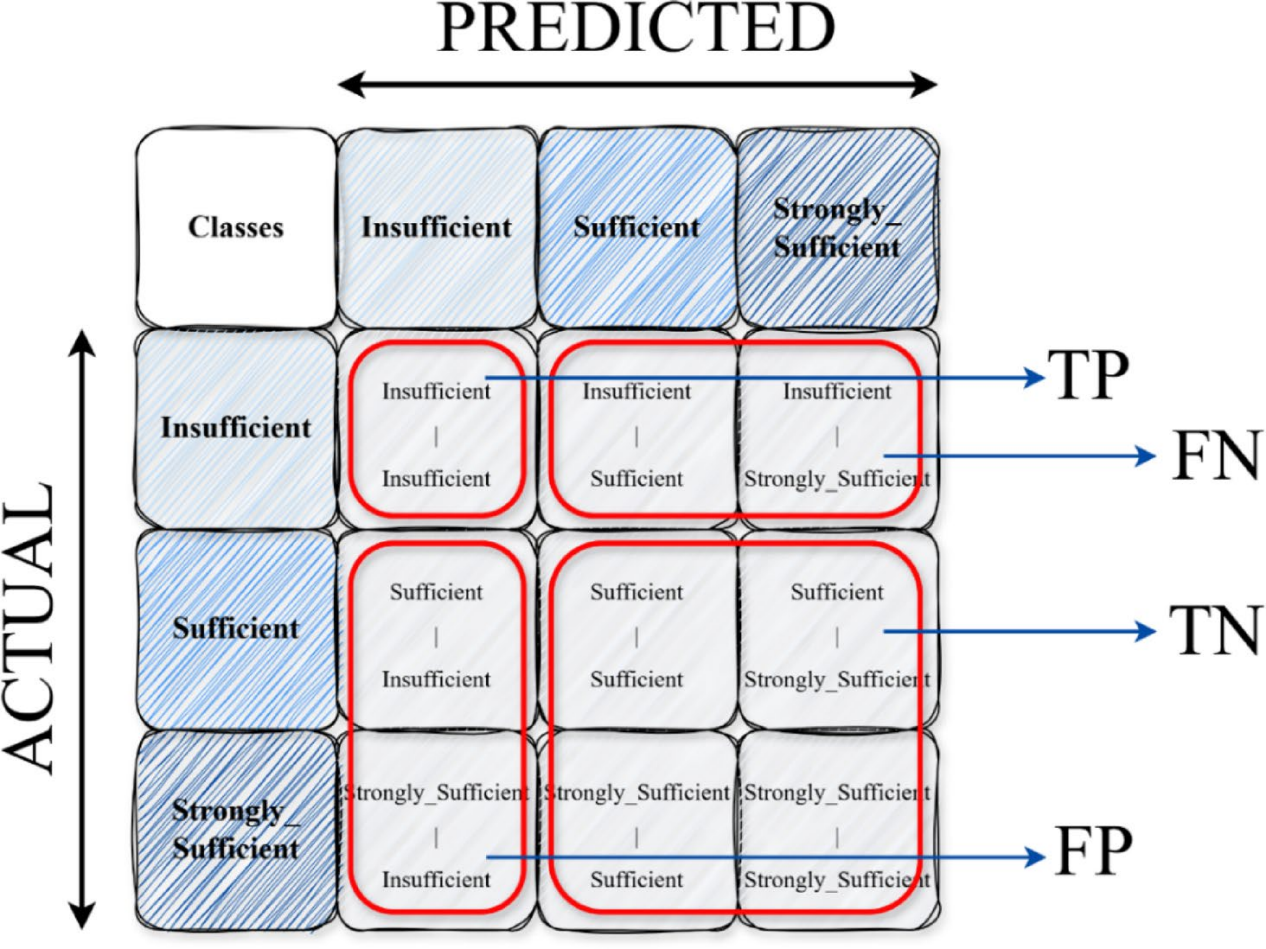
Confusion matrix display 3 × 3.

The confusion matrix is a fundamental tool for visualizing the performance of classification models. In multi-class problems (3 classes in this study), the matrix is created as an N×N table (N: number of classes). One axis represents the true classes, the other the classes predicted by the model. Values on the diagonal of the matrix represent the number of correct predictions for each class^22^. Values outside the diagonal reveal which classes the model confused with each other, i.e., the types of errors it made^23^. This structure allows one to understand the model’s strengths and weaknesses at a glance. Figure 3 presents a 3 × 3 confusion matrix.

*Accuracy* indicates the proportion of correct predictions made by the model. It is calculated by dividing the sum of the diagonal values in the confusion matrix by the total number of samples^24^. While a general indicator of success, it can be misleading on its own in datasets with imbalanced class distributions^25^. The calculation for accuracy can be obtained from formula (1).

$$\:Accuracy=\:\frac{{\sum\:}_{i=1}^{K}{TP}_{i}}{Total\:Number\:of\:Samples}\times\:100\:\:\:\:\:\:\:\:\:\:\:\:\:\:\:\:\:\:\:\:\:\:\:\:\:\:\:\:\:\:\:\:\:\:\:\:\:\:\:\:\:\:\:\:\:\:\:\:\:\:\:\:\:\:\:\:\:\:\:\:\:\:\:\:\:\:\:\:\:\:\:\:\:\:\:\:\:\:\:\:\:\:\:\:\:\:\:\:\:\left(1\right)$$

In multiclass classification, these metrics are usually calculated separately for each class. When calculating, one class is considered “positive” and all other classes are considered “negative”^26^.

*Precision m*easures how many of the positive predictions for a class are actually correct. This is important when false positive alarms are costly^23^. The calculation for precision can be obtained from formula (2).

$$\:Precision=\:\frac{{\sum\:}_{i=1}^{K}\frac{{TP}_{i}}{{TP}_{i}+{FP}_{i}}}{Total\:Number\:of\:Samples}\times\:100\:\:\:\:\:\:\:\:\:\:\:\:\:\:\:\:\:\:\:\:\:\:\:\:\:\:\:\:\:\:\:\:\:\:\:\:\:\:\:\:\:\:\:\:\:\:\:\:\:\:\:\:\:\:\:\:\:\:\:\:\:\:\:\:\:\:\:\:\:\:\:\:\:\:\:\:\:\:\:\:\:\:\:\:\:\:\:\:\left(2\right)$$

*Sensitivity (Recall)* indicates how many examples of a class were correctly detected by the model. It is a priority in situations where missing positive cases is critical^27^. The calculation for recall can be obtained from formula (3).

$$\:Recall=\:\frac{{\sum\:}_{i=1}^{K}\frac{{TP}_{i}}{{TP}_{i}+{FN}_{i}}}{Total\:Number\:of\:Samples}\times\:100\:\:\:\:\:\:\:\:\:\:\:\:\:\:\:\:\:\:\:\:\:\:\:\:\:\:\:\:\:\:\:\:\:\:\:\:\:\:\:\:\:\:\:\:\:\:\:\:\:\:\:\:\:\:\:\:\:\:\:\:\:\:\:\:\:\:\:\:\:\:\:\:\:\:\:\:\:\:\:\:\:\:\:\:\:\:\:\:\:\:\:\:\:\:\:\left(3\right)$$

*F1-Score* is the harmonic mean of the precision and sensitivity metrics. It summarizes the overall success of the model by establishing a balance between these two metrics and is a reliable metric, especially in unbalanced datasets^27^. The calculation for F1-score can be obtained from formula (4).

$$\:F1-Score=\:2\times\:\frac{Precision\:\times\:Recall}{Precision+Recall}\times\:100\:\:\:\:\:\:\:\:\:\:\:\:\:\:\:\:\:\:\:\:\:\:\:\:\:\:\:\:\:\:\:\:\:\:\:\:\:\:\:\:\:\:\:\:\:\:\:\:\:\:\:\:\:\:\:\:\:\:\:\:\:\:\:\:\:\:\:\:\:\:\:\:\:\:\:\:\:\:\:\:\:\:\:\:\:\:\:\:\:\:\:\:\left(4\right)$$

### Cross-validation

Cross-validation is a widely used technique to reduce the risk of overfitting and to evaluate model performance more reliably. In this approach, the dataset is divided into K equal subsets (folds). At each iteration, one fold is used as the test set while the model is trained on the remaining K–1 folds, and this process is repeated K times so that each fold serves once as the test set^28^. By averaging the results across folds, a more robust estimate of the model’s generalization performance is obtained^29^. In the present study, stratified 5-fold cross-validation was applied, ensuring that class proportions were preserved in each fold. To further mitigate overfitting risks, all models were evaluated under this scheme and additionally tested on hold-out data partitions. Algorithm-specific mechanisms also acted as implicit regularization: for instance, Logistic Regression and MLP applied tuned regularization parameters (C, α), while tree-based methods (Decision Tree, Random Forest, Gradient Boosting, XGBoost) were constrained by maximum depth and minimum samples per split/leaf to avoid overly complex models. With respect to class distribution, the dataset contained 101 “Insufficient,” 298 “Sufficient,” and 217 “Strongly Sufficient” cases, corresponding to a smallest-to-largest ratio of approximately 1:3. This level of imbalance is considered manageable in classification tasks^30^, and therefore no re-sampling was required. Model reliability was further supported by the low variance of performance metrics across folds, confirming that the findings are stable and not driven by chance data splits.

### Data mining methods

This sub-section provides a detailed overview of the core data mining methods employed in the study. The theoretical foundations, learning mechanisms, and relevant literature for each model are presented to explain how these methods contribute to solving classification problems. Accordingly, the rationale behind model selection is clarified, laying the groundwork for the experimental results discussed in the subsequent sections.

### Support vector machines (SVM)

SVM is a powerful and flexible supervised learning algorithm that provides effective results in both classification and regression problems^31^. The main goal of SVM is to determine an optimal hyperplane that best separates the data and maximizes the margin between classes. This margin is defined by the support vectors, which are the data points closest to the decision boundary, and plays a critical role in increasing the generalizability of the model^32^. Because SVM creates the decision function using only a small subset of training data called support vectors, it demonstrates high performance especially on small and medium-sized complex datasets^33^. This approach, which reduces the risk of overfitting by controlling model complexity, is based on the principle of structural risk minimization^34^. Owing to these features, SVM; it is widely used in a wide variety of fields such as image recognition^35^, text classification, bioinformatics and financial forecasting^36^.

### Artificial neural networks (ANN)

ANNs are computational models inspired by the human brain’s neural structure, offering strong capabilities for modeling complex, nonlinear patterns^37^. They consist of an input layer, one or more hidden layers, and an output layer^38^, with artificial neurons connected by adjustable weighted links^28^. Data flows forward through the layers, where each neuron computes a weighted sum of inputs, adds a bias, and applies an activation function to produce output^39^, introducing nonlinearity and enhancing learning capacity^40^. ANN architectures influence learning and generalization, and training is typically carried out via the backpropagation algorithm^41^. Their flexibility and strong performance make ANNs highly effective for classification, prediction, and pattern recognition tasks on complex datasets.

### Random forest (RF)

RF is an ensemble learning method used effectively in solving both classification and regression problems. This algorithm is based on the construction of multiple decision trees, each trained with randomly selected subsamples (bootstraps) from the original dataset^42^. This randomness among trees increases model diversity, reduces overfitting, and improves overall performance. Random Forest produces more reliable and widely applicable outcomes by combining the outputs from multiple decision trees. The algorithm demonstrates particularly strong performance in analyzing high-dimensional and nonlinear data structures. It achieves its results by majority voting in classification problems and by averaging the predictions obtained from the trees in regression problems^43^.

### k-Nearest neighbors (k-NN)

The k-NN algorithm offers a flexible approach to both classification and regression problems in supervised machine learning. Owing to its parameter-free structure, it does not require a specific training process; instead, it stores all training data and relies on the proximity principle when making predictions about new examples^44^. Predictions are made using the k nearest neighbors, typically determined by a suitable metric such as the Euclidean distance^45^. The algorithm predicts new data using the most common class in classification and the average of neighbors in regression. The selection of the k value is critical to the model’s performance; low values can lead to overfitting, while high values can cause the model to miss complex patterns^46^.

### Logistic regression (LR)

Despite the word “regression” in its name, LR is a fundamental supervised learning algorithm widely used in classification problems and can be applied to both binary and multiclass situations. This method uses the logistic (sigmoid) function to model the probability that an observation belongs to a particular category. The logistic function provides a probabilistic interpretation in classification problems by limiting the output of the linear model between 0 and 1^47^. The key difference of logistic regression is that, unlike linear regression, which estimates continuous numerical values, it models categorical outcomes. The maximum likelihood method is used for parameter estimation^48^. The explanatory power of this model and its broad range of applications make it particularly preferred in medicine, marketing, and the social sciences^49^.

### Decision tree (DT)

DT algorithms offer flexible and effective solutions for both classification and regression tasks in supervised learning. Data is recursively partitioned based on its characteristics, with decision rules located at each internal node, and final predictions located at leaf nodes^50^. The goal is to increase prediction accuracy by partitioning the data in a way that best aligns with the target variable^51^. The key advantages of DTs include easy interpretability, the ability to work with numerical and categorical data, a visualizable structure, and more reliable against missing/outlier values^52^. These features make them a practical and reliable option when working with complex datasets.

### AdaBoost

AdaBoost (Adaptive Boosting) is an effective machine learning method that combines weak classifiers into an ensemble structure to improve performance. The algorithm updates the weights of training examples at each iteration, focusing more on misclassified examples^53^. Thanks to this sequential structure, each new model attempts to correct previous errors, thereby improving accuracy^43^. AdaBoost is often used with simple algorithms such as decision trees and achieves high success in classification tasks. It can be sensitive to noisy data; however, with appropriate data preprocessing, it can produce results resilient to overfitting^54^. Boosting generally aims to transform multiple weak classifiers into a strong model by identifying problematic examples and assigning them more weight^55^. The effect of weights used in the sampling or modeling process affects model performance through variance and bias reduction, respectively^56^.

### Gradient boosting

Gradient Boosting is an ensemble learning method whereby weak learners (typically small decision trees) are trained sequentially, with each new model aiming to correct the errors of previous models. The model is updated using the gradient of the loss function, thereby improving prediction accuracy. This method is widely used in both classification and regression problems, but training time can be long and requires careful hyperparameter tuning to prevent overfitting^43^. Boosting algorithms transform weak classifiers into stronger models by weighting misclassified examples^55^. Gradient Boosting can also be enhanced with techniques such as Newton updates to optimize model parameters^57^ and adapted to different applications^58^. This sequential structure allows subsequent classifiers to focus on difficult examples, and the final predictions are obtained by summing the weighted results of all trees^59^.

### XGBoost

XGBoost is a gradient boosting algorithm optimized for speed and performance that generates an ensemble of sequential decision trees by optimizing a regularized objective function^60^. It uses regularization techniques to control noise and prevent overfitting. Its parallel operation with multi-core processors and efficient handling of large datasets make it widely preferred in classification and regression^61^. XGBoost automatically handles missing values, evaluates feature importance, and demonstrates more reliable performance on balanced and unbalanced datasets. It is recognized for its high accuracy and speed in both academia and industry. The flexible adjustment of model parameters enables it to be adapted to different applications^62^. New models strengthen the ensemble by estimating the errors of previous models, and the final prediction is made by summing the outputs of all models^63^.

### Heatmap

A heatmap is an analysis tool that visualizes numerical data based on color intensity. In assessing 21 st century competencies, students’ performance levels in skills such as critical thinking, problem solving, collaboration, and digital literacy can be easily compared using a heatmap. Color tones are assigned meanings based on proficiency levels; darker colors represent higher achievement, and lighter colors represent lower achievement. This visualization allows for quick identification of strengths and weaknesses, contributes to curriculum mapping, and can reveal previously unnoticed data patterns^64^. Furthermore, when used on student dashboards, it supports self-regulated learning, allowing students to monitor their own learning^65^.

### Grid search

Grid Search is a systematic search method used to find the optimal hyperparameter combinations in machine learning models. The validation performance of the model is evaluated by trying all possible combinations based on the specified hyperparameter values. This method is particularly important for improving performance in complex models and achieving a balance between overfitting and underfitting^66^. Although Grid Search is computationally intensive and time-consuming, it is widely preferred due to the reliability and reproducibility it provides on model performance^67^. While alternative methods (e.g., random search and Bayesian optimization) can provide faster results, Grid Search’s comprehensive scanning feature provides an advantage for fully exploring hyperparameter settings^68^. In multidimensional and complex training data, such as 21 st century competencies, models optimized with Grid Search increase classification accuracy, contributing to educators’ data-driven decision-making processes.

The hyperparameter grids used in the GridSearchCV were carefully defined for each model. For Logistic Regression, the LBFGS solver with a maximum of 1000 iterations was applied, with the regularization parameter $\:C\:$tested at values {0.01, 0.1, 1, 10} under an L2 penalty. For Random Forest, the search space included *n_estimators* $\:\in\:${100, 200}, *max_depth* $\:\in\:${None, 10, 20}, *min_samples_split* $\:\in\:${2, 5}, and *min_samples_leaf* $\:\in\:${1, 2}. The Decision Tree model was tuned with the same ranges for maximum depth, minimum split, and minimum leaf size. For the Support Vector Machine with an RBF kernel, the hyperparameters explored were $\:C$ $\:\in\:$ {0.1, 1, 10} and γ ∈ {scale, auto}. In the case of k-Nearest Neighbors, the number of neighbors was set to {3, 5, 7}, with weights either uniform or distance-based. For AdaBoost, the grid included *n_estimators* $\:\in\:$ {50, 100} and *learning_rate* ∈ {0.5, 1.0, 1.5}. Gradient Boosting was tuned with *n_estimators* ∈ {100, 200}, *learning_rate* ∈ {0.05, 0.1, 0.2}, and *max_depth* ∈ {3, 5, 7}. Similarly, for XGBoost (configured with *use_label_encoder* = False and *eval_metric*=‘*mlogloss*’), the grid consisted of the same ranges for *n_estimators*, learning rate, and maximum depth. The Artificial Neural Network (MLP) was optimized with hidden layer sizes (50), (100), (100, 50), activation functions {relu, tanh}, the Adam solver, α ∈ {0.0001, 0.001, 0.01}, and learning rate options {constant, adaptive}, with a maximum of 1000 iterations. All models were implemented and optimized in a Python 3.0 environment using scikit-learn, XGBoost (XGBClassifier with *use_label_encoder* = False, eval_metric=‘mlogloss’), NumPy, and pandas.

## Experimental results and discussion

This section presents the experimental results derived from the analysis of survey data, structured according to four distinct sub-dimensions and a combined dataset. The analyses for each section consistently involved data loading, preprocessing, and dimensionality reduction for visualization purposes. For each sub-dimension and the combined dataset, the experimental process began by loading the respective Excel file. The features used for analysis consisted of the Likert scale items relevant to each sub-dimension, while the ‘CLASS’ column served as the target variable for potential classification tasks.

###  21 st century competencies knowledge subscale

**Fig. 4:**
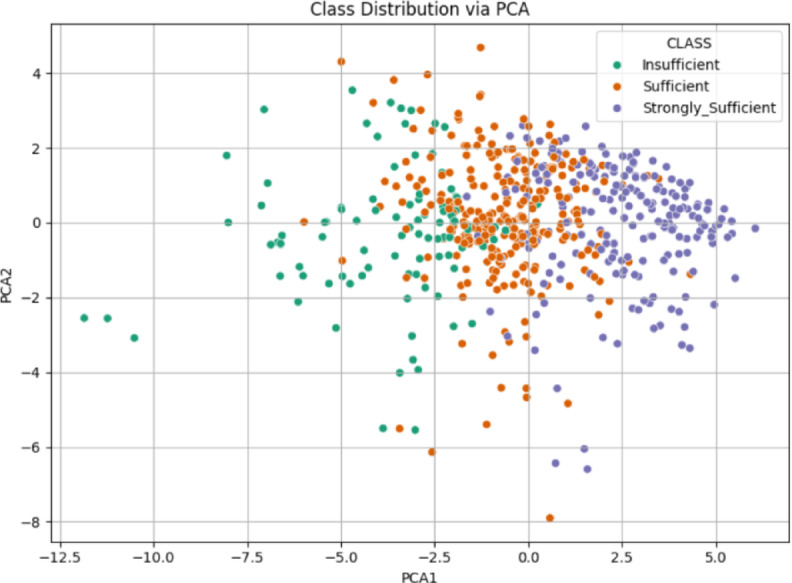
Class distribution via PCA scatter plot for knowledge sub-dimension.

The analysis for the “Knowledge Sub-Dimension” used data from and Excel file. After loading, the relevant Likert items were selected as features, and the ‘CLASS’ column was identified as the target. Data was scaled using StandardScaler, and PCA was performed to reduce the features to two principal components. The scatter plot in Fig. 4 illustrates the class distribution of the Knowledge Sub-Dimension based on the first two principal components obtained through PCA. The insufficient and Strongly Sufficient classes are relatively well-separated, primarily occupying the negative and positive regions of PCA1, respectively. In contrast, the sufficient class is widely dispersed and overlaps with both other groups, particularly in the central area. This overlap suggests limited separability between classes, indicating potential classification challenges in this feature space.

**Fig. 5:**
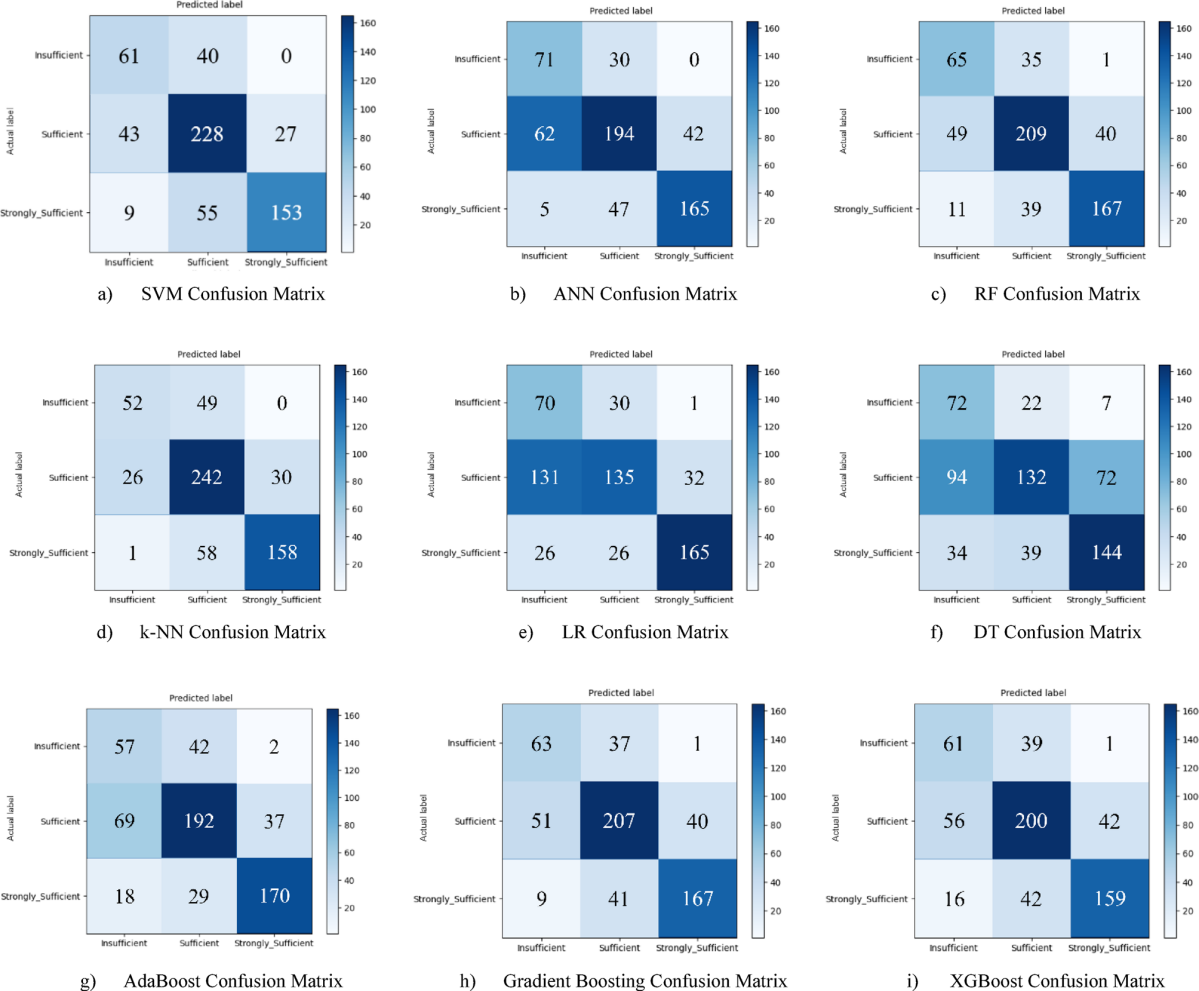
Sub-dimension (21st century competencies knowledge sub-scale) confusion matrix.

**Fig. 6:**
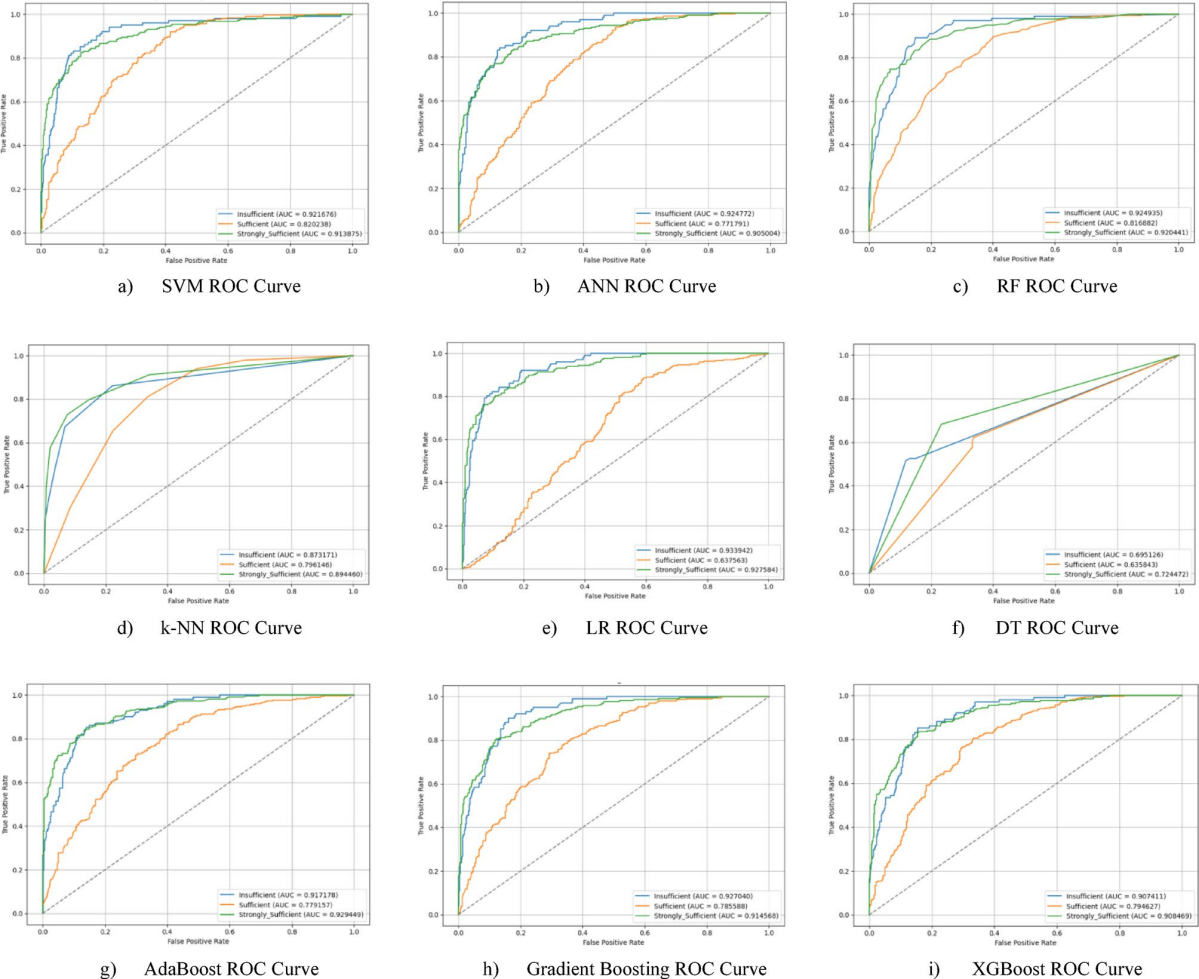
Sub-dimension (21st century competencies knowledge sub-Scale) ROC curves.

Nine different machine learning models were tested for the first subscale of this study, the “21st Century Competencies Knowledge Subscale.” The results showed that the k-NN model achieved the highest classification success with an accuracy rate of 73.4%. The k-NN model performed notably well, particularly in the (sufficient) and (strongly sufficient) classes, with high F1 scores of 0.748 and 0.780, respectively, significantly boosting the model’s overall effectiveness. The confusion matrix and ROC curves of the k-NN model are presented in Fig. 5(d) and Fig. 6(d), respectively.

The SVM and RF models followed closely behind k-NN, achieving accuracy scores of 71.8% and 71.6%, respectively. Both of these models demonstrated balanced and high performance across all classes. The confusion matrix of the SVM is shown in Fig. 5(a), and the ROC curve is shown in Fig. 6(a). Similarly, the results of the RF model can be seen in Fig. 5(c) and Fig. 6(c). On the other hand, the LR and DT models exhibited the lowest performance for this sub-dimension, with accuracy rates of 60.1% and 56.5%, respectively, shown in Figs. 5(e, f) and Figs. 6(e, f). Their low precision, particularly in predicting the insufficient class, negatively impacted the overall performance of these models. Other models examined, AdaBoost, Gradient Boosting, XGBoost, and ANN, demonstrated moderate success rates ranging from 68% to 71%. Detailed results and related images for these models are presented in Fig. 5(g-i) and 6(g-i).

The ROC curves for all nine classification models used in evaluating the Knowledge Sub-Dimension of 21 st Century Competencies are presented in Fig. 6(a-i). These plots illustrate each model’s performance across the three class categories.

Comparative results of the models are presented in Table 2. This table clearly demonstrates that the k-NN model is the most suitable model, demonstrating the highest success across all metrics. The SVM and RF models also produced very similar and successful results.

**Table 2:** Model performance comparison table for knowledge Sub-Scale.

Model	Accuracy (%)	Precision (%)	Recall (%)	F1-Score (%)
SVM	71.75	72.94	71.75	72.02
ANN	69.81	71.15	69.81	70.15
RF	71.59	72.54	71.59	71.92
k-NN	73.38	73.94	73.38	73.15
LR	60.06	68.61	60.06	61.72
DT	56.49	61.74	56.49	56.91
AdaBoost	68.02	70.46	68.02	68.86
Gradient Boosting	70.94	71.82	70.94	71.26
XGBoost	68.18	69.68	68.18	68.70

**Fig. 7:**
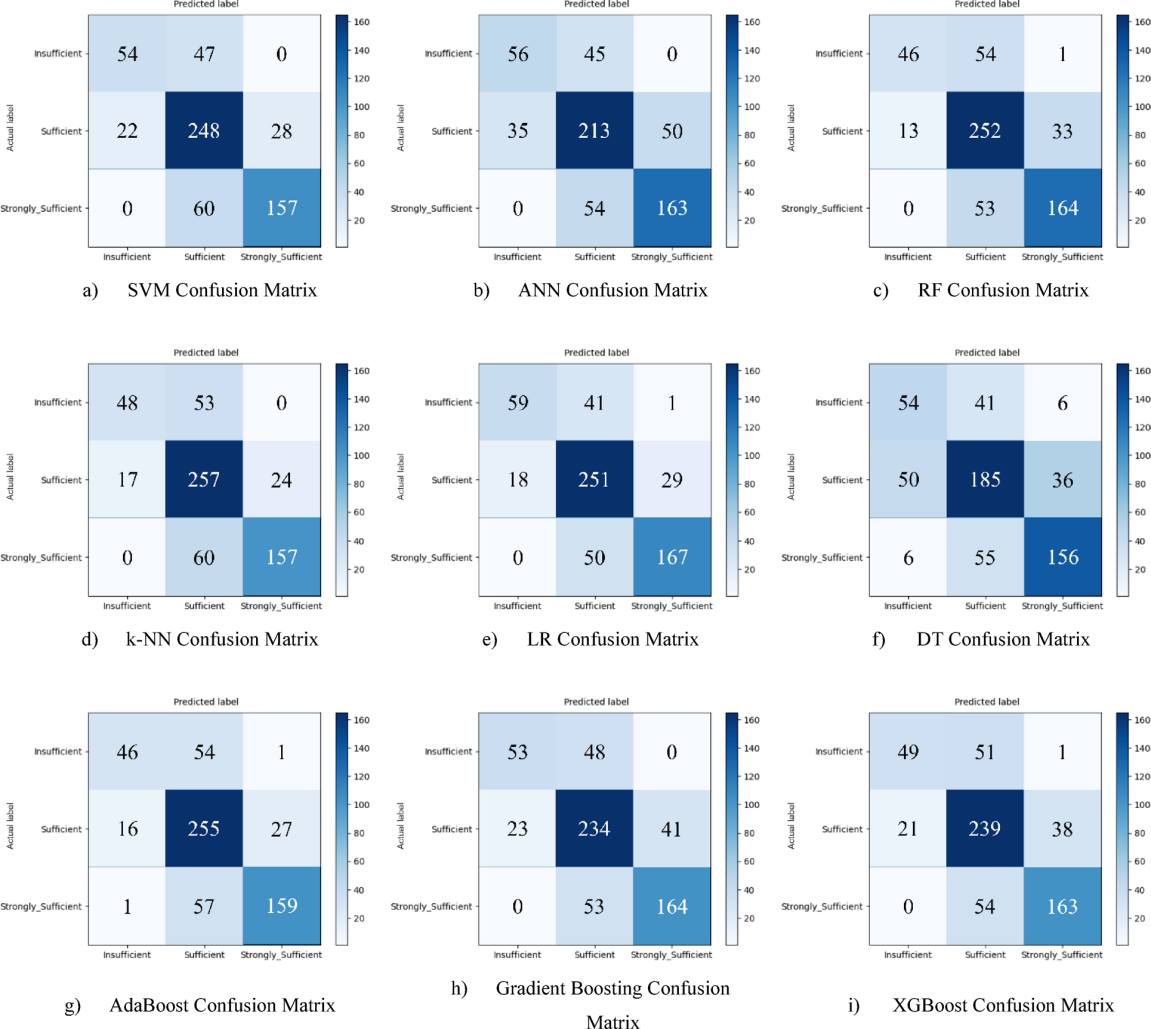
Sub-dimension (21st century competencies knowledge sub-scale) grid search optimization confusion matrix.

**Fig. 8:**
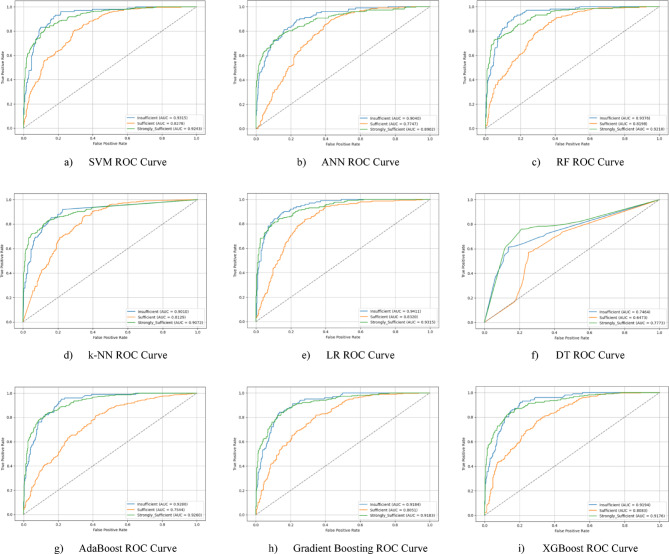
Sub-dimension (21st century competencies knowledge sub-scale) ROC curves.

Hyperparameter optimization was performed using the Grid Search method to improve the performance of the classification models. At the end of this optimization process, it was observed that all models achieved higher performance compared to the baseline results. After optimization, the LR model achieved the highest classification accuracy with 77.4%. This success was achieved with the parameters {clf__C: 0.1, clf__penalty: ‘l2’, clf__solver: ‘lbfgs’}. The confusion matrix of the optimized LR model is presented in Fig. 7(e), and the ROC curve is presented in Fig. 8(e). The LR model was followed by the RF and k-NN models, which achieved accuracies of 75.5% and 75.0%, respectively. Other models (SVM, AdaBoost, ANN, Gradient Boosting, XGBoost) were also found to produce highly competitive results in the 73%−75% range. This process demonstrates how appropriate hyperparameter settings positively impact model performance. Performance graphs (confusion matrix and ROC curve) for each optimized model are presented in the corresponding figures (Fig. 7(a-i) and 8(a-i)).

The ROC curves for all nine classification models used in evaluating the Knowledge Sub-Dimension of 21 st Century Competencies with grid search optimization are presented in Fig. 8(a-i). These plots illustrate each model’s performance across the three class categories.

The results of hyperparameter optimization performed using Grid Search for each machine learning model are summarized in Table 3. This table presents the best classification accuracy obtained (%) for the Knowledge Sub-Scale, along with the corresponding optimal parameter settings identified during the tuning process.

**Table 3:** Grid search optimization results and optimal parameters for knowledge sub-scale.

Model	Best Accuracy (%)	Best Parameters
SVM	74.51	C: 1, gamma: scale, kernel: rbf
ANN	74.36	activation: relu, alpha: 0.0001, hidden_layer_sizes: (100, 50)
RF	75.49	n_estimators: 200, max_depth: 10, min_samples_leaf: 2
k-NN	75.01	n_neighbors: 7, weights: distance
LR	77.44	C: 0.1, penalty: l2, solver: lbfgs
DT	65.10	max_depth: 10, min_samples_leaf: 2, min_samples_split: 5
AdaBoost	74.68	n_estimators: 100, learning_rate: 0.5
Gradient Boosting	73.21	n_estimators: 100, learning_rate: 0.05, max_depth: 3
XGBoost	73.21	n_estimators: 100, learning_rate: 0.05, max_depth: 3

**Fig. 9:**
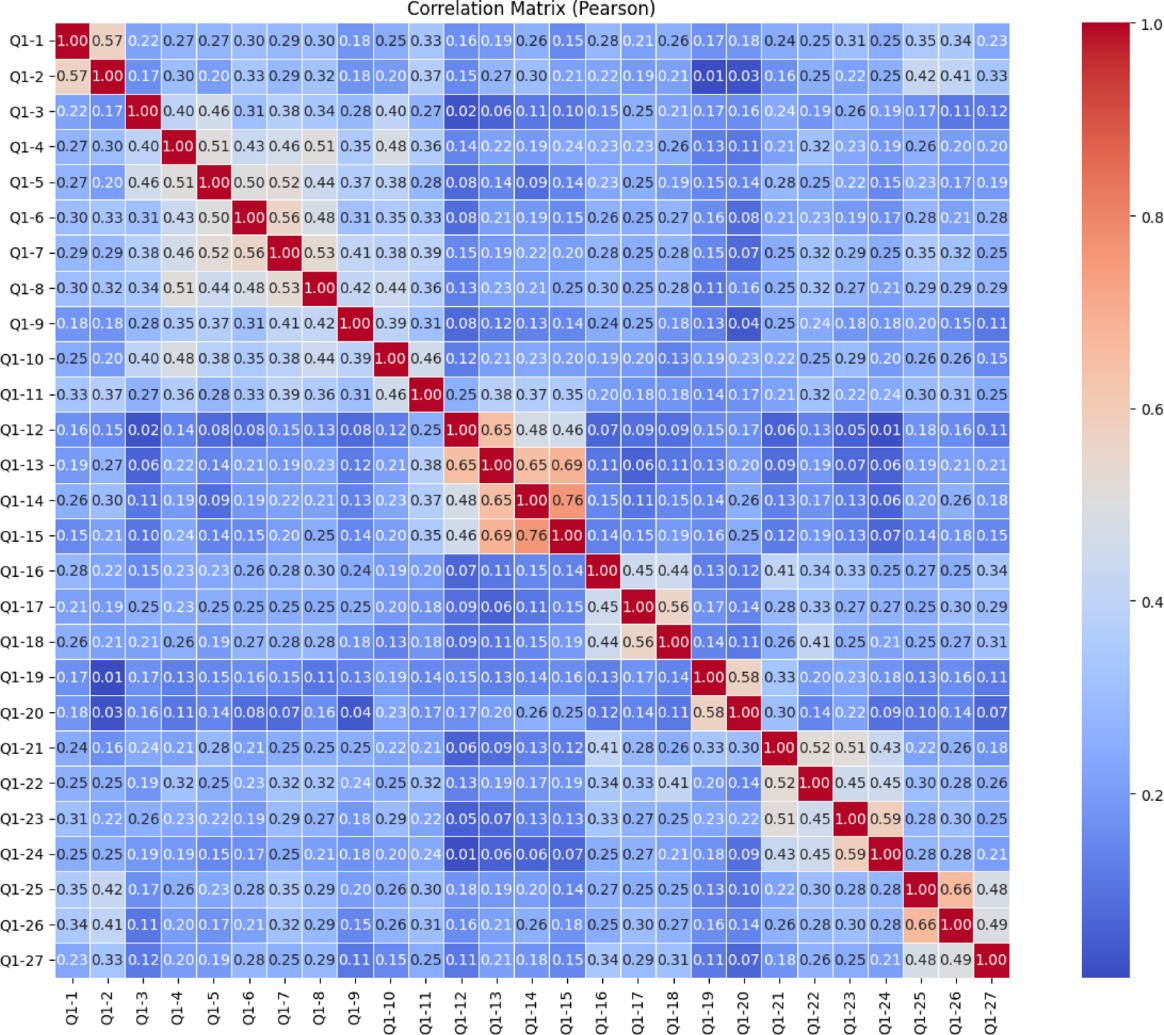
Knowledge sub-dimension pearson correlation heatmap.

The heatmap presented in the image (Fig. 9) shows Pearson correlation relationships between 27 different variable survey items named from Q1-1 to Q1-27. The Pearson correlation matrix for the Knowledge Sub-Dimension, which includes items Q1-1 through Q1-27, indicates that the majority of item relationships fall within the low to moderate range. Most correlation values range between 0.10 and 0.40, reflecting generally weak linear associations among the items. This pattern suggests that while the items are likely aligned under the broader construct of knowledge, they may be measuring different, more specific aspects of it. However, a few notable clusters of stronger correlations are evident. In particular, Q1-13, Q1-14, and Q1-15 show relatively high correlations with each other, with values reaching up to 0.76. Similarly, Q1-25, Q1-26, and Q1-27 also display strong correlations, indicating potential sub-dimensions or closely related content areas within the scale. These clusters suggest that some items may form internally consistent groupings, while others remain more independent. Overall, the matrix points to a likely multidimensional structure of the Knowledge Sub-Dimension, and further statistical procedures, such as exploratory or confirmatory factor analysis, are recommended to clarify the underlying components and optimize the scale’s structure.

###  21 st century competencies skills subscale

**Fig. 10:**
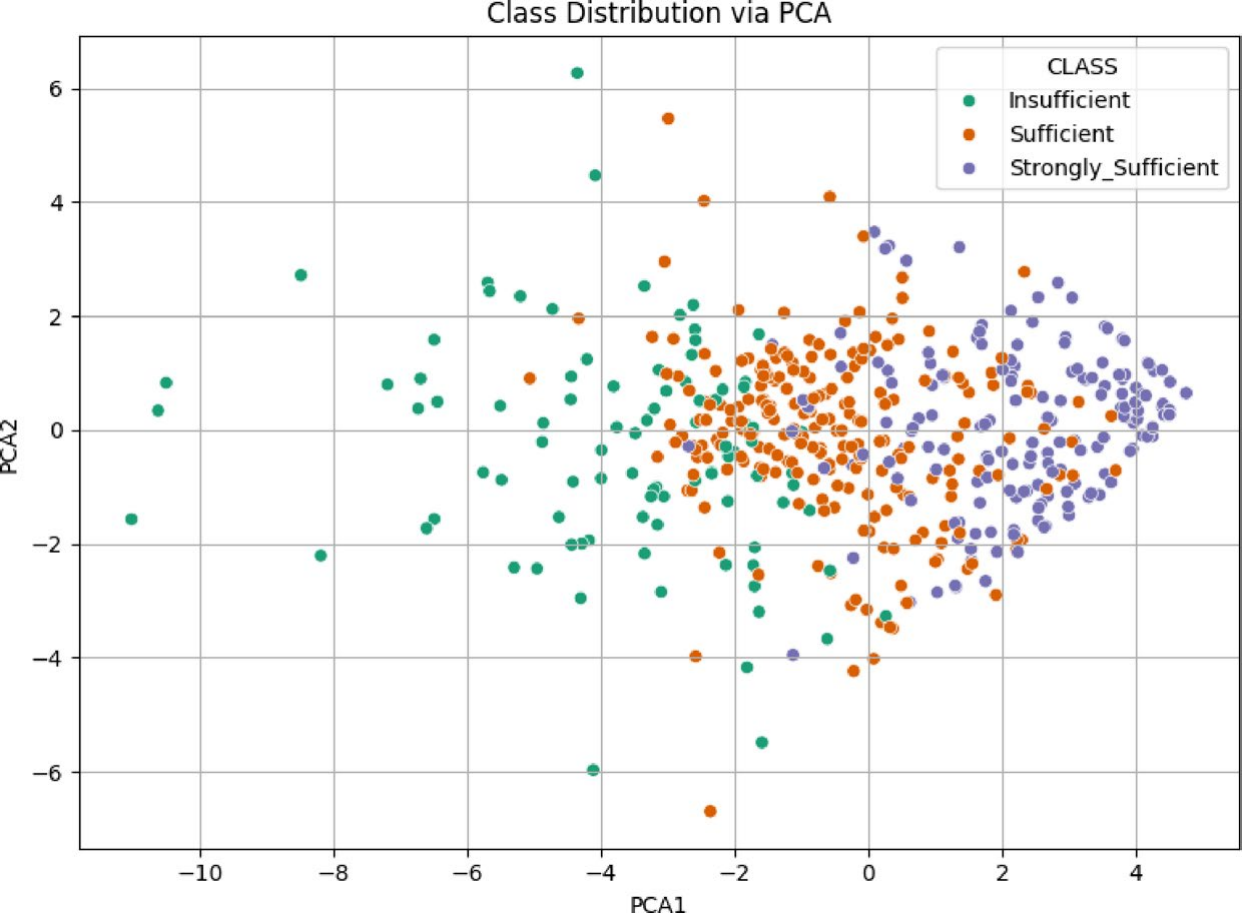
Class distribution via PCA scatter plot for skills sub-dimension.

The scatter plot in Fig. 10 illustrates the class distribution of the 21 st Century Competencies Skills Sub-Dimension using the first two principal components derived from PCA. The Insufficient class tends to cluster on the far left (negative PCA1), while the Strongly Sufficient group is more concentrated on the far right (positive PCA1). The Sufficient class is again spread between the two, with a noticeable overlap with both extremes. Although a general separation pattern is visible (particularly between Insufficient and Strongly Sufficient), the intermediate class remains intermingled, indicating moderate class overlap and suggesting a gradual transition in competency levels across the data.

**Fig. 11:**
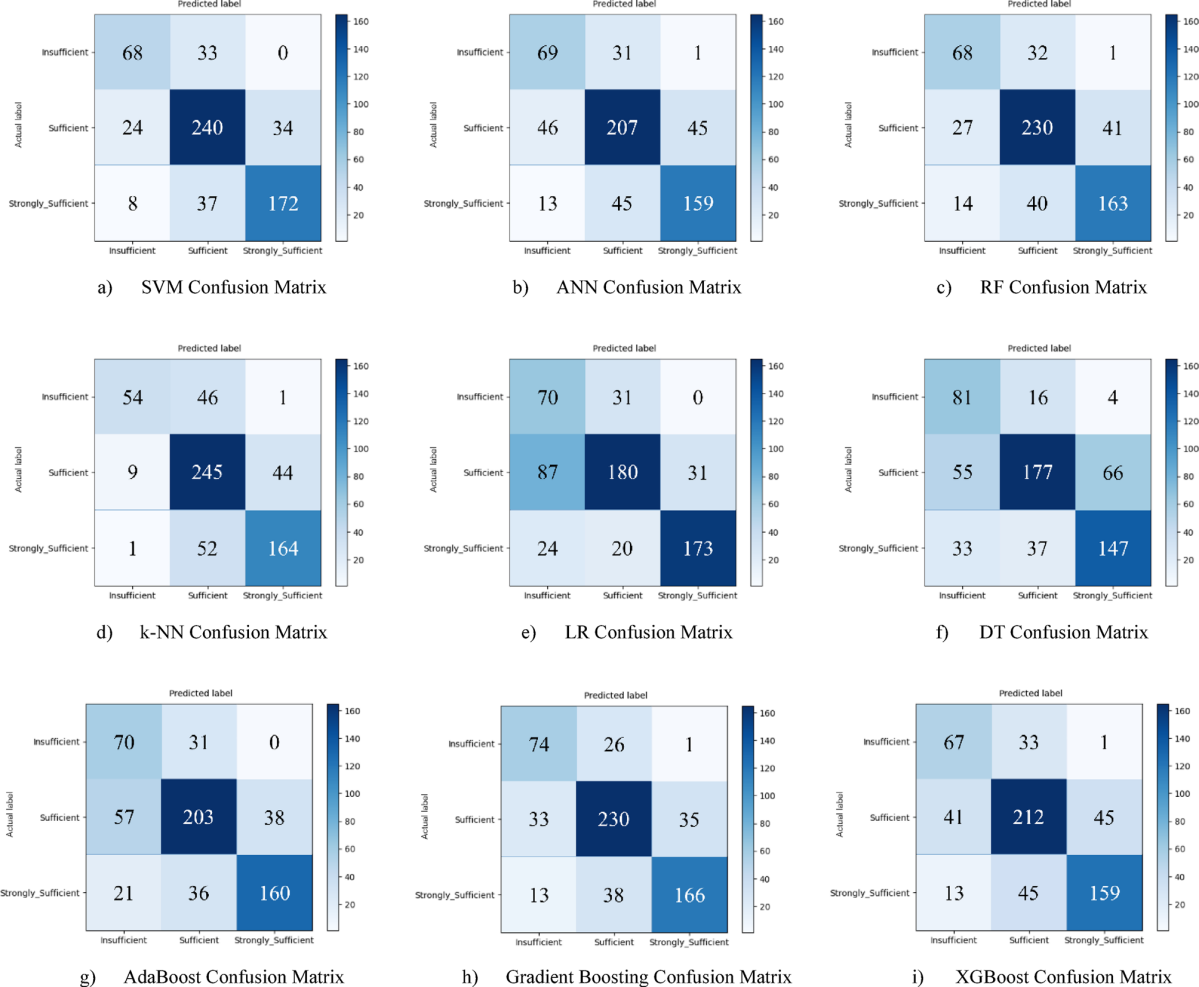
Sub-dimension (21st century competencies skills subscale) confusion matrix.

In the initial modeling for the “21st Century Competencies Skills Subscale”, the SVM model showed the highest classification success with an accuracy rate of 77.9%. The fact that SVM achieved balanced and high F1 scores in all classes forms the basis of this success. Gradient Boosting (76.3%), k-NN (75.2%), and RF models (74.8%) followed SVM with very competitive results, respectively. LR and especially Decision Tree models performed less well in this sub-dimension compared to the others. The confusion matrix and ROC curve of each model are presented in the relevant figures (Fig. 11(a-i)).

**Fig. 12:**
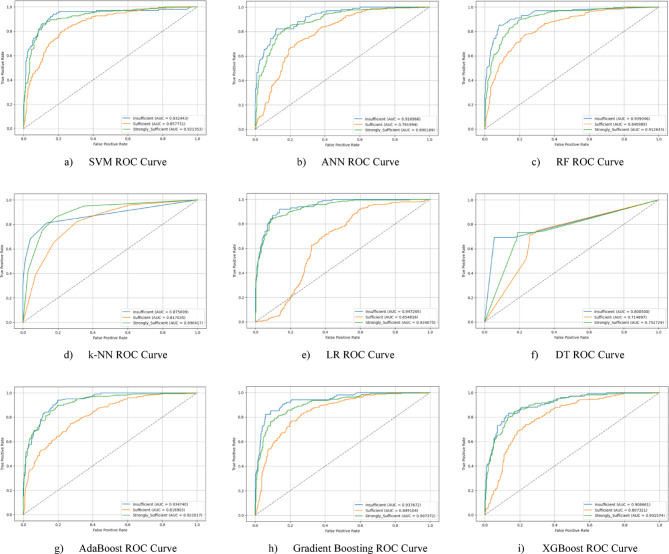
Sub-dimension (21st century competencies skills sub-scale) ROC curves.

The ROC curves for all nine classification models used in evaluating the Skills Sub-Dimension of 21 st Century Competencies with grid search optimization are presented in Fig. 12(a-i). These plots illustrate each model’s performance across the three class categories.

Comparative results of the models are presented in Table 4. This table clearly demonstrates that the SVM model is the most suitable model, demonstrating the highest success across all metrics. Gradient Boosting and k-NN models also produced very similar and successful results.

**Table 4:** Model performance comparison table for skills Sub-Scale.

Model	Accuracy (%)	Precision (%)	Recall (%)	F1-Score (%)
SVM	77.92	78.02	77.92	77.93
ANN	70.62	71.55	70.62	70.90
RF	74.84	75.08	74.84	74.92
k-NN	75.16	76.03	75.16	74.84
LR	68.67	73.91	68.67	70.01
DT	65.75	68.95	65.75	66.14
AdaBoost	70.29	72.59	70.29	70.96
Gradient Boosting	76.30	76.91	76.30	76.48
XGBoost	71.11	71.77	71.10	70.90

**Fig. 13:**
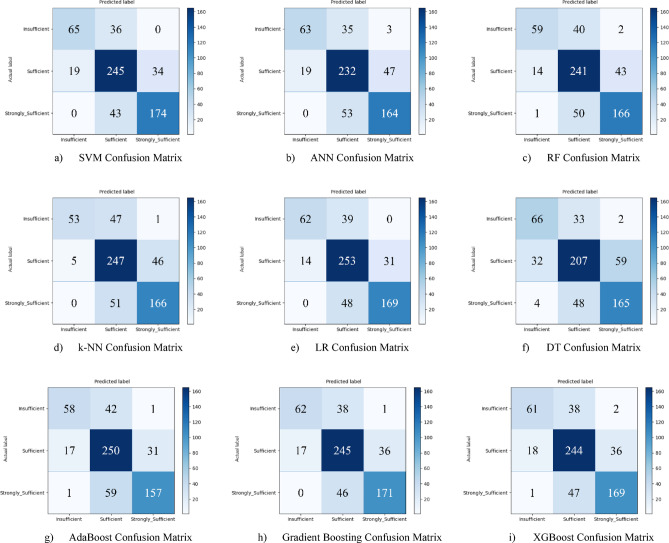
Sub-dimension (21st century competencies skills subscale) grid search optimization confusion matrix.

Grid Search hyperparameter optimization, performed to maximize model performance, significantly increased overall performance. At the end of the optimization process, the LR and SVM models achieved the highest and equal accuracy with 78.6%, becoming the best models in this sub-dimension. This result is significant because it demonstrates how effective the LR model, which initially performed less well, can be with the right parameters. The RF (77.4%), Gradient Boosting (77.0%), and XGBoost (77.0%) models also proved to be quite successful on this dataset, achieving results very close to the best models. Confusion matrix and ROC curves are presented in Fig. 13(a-i).

**Fig. 14:**
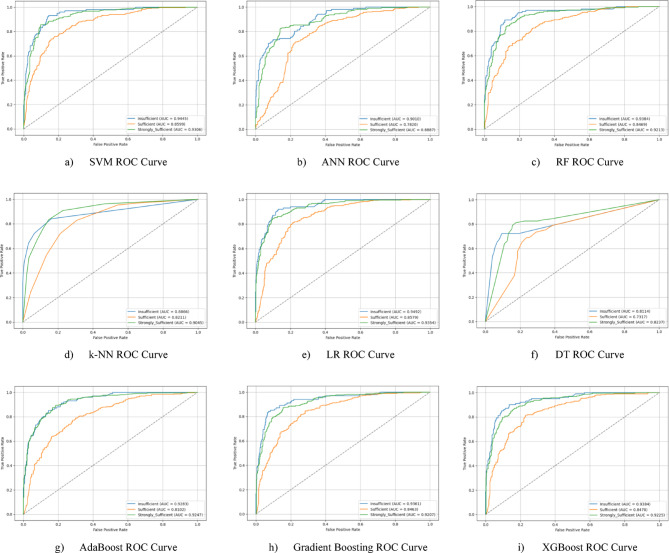
Sub-dimension (21st century competencies skills sub-scale) grid search optimization ROC curves.

The ROC curves for all nine classification models used in evaluating the Skills Sub-Dimension of 21 st Century Competencies with grid search optimization are presented in Fig. 14(a-i). These plots illustrate each model’s performance across the three class categories.

The results of hyperparameter optimization performed using Grid Search for each machine learning model are summarized in Table 5. This table presents the best classification accuracy obtained (%) for the Skills Sub-Scale, along with the corresponding optimal parameter settings identified during the tuning process.

**Table 5:** Grid search optimization results and optimal parameters for skills Sub-Scale.

Model	Best Accuracy (%)	Best Parameters
SVM	78.58	C: 1, gamma: scale, kernel: rbf
ANN	75.00	activation: relu, alpha: 0.001, hidden_layer_sizes: (50,)
RF	77.44	n_estimators: 100, max_depth: 20, min_samples_leaf: 1
k-NN	75.49	n_neighbors: 7, weights: uniform
LR	78.58	C: 0.1, penalty: l2, solver: lbfgs
DT	72.40	max_depth: 20, min_samples_leaf: 1, min_samples_split: 5
AdaBoost	75.49	n_estimators: 50, learning_rate: 0.5
Gradient Boosting	76.95	n_estimators: 100, learning_rate: 0.05, max_depth: 3
XGBoost	76.95	n_estimators: 100, learning_rate: 0.1, max_depth: 3

**Fig. 15:**
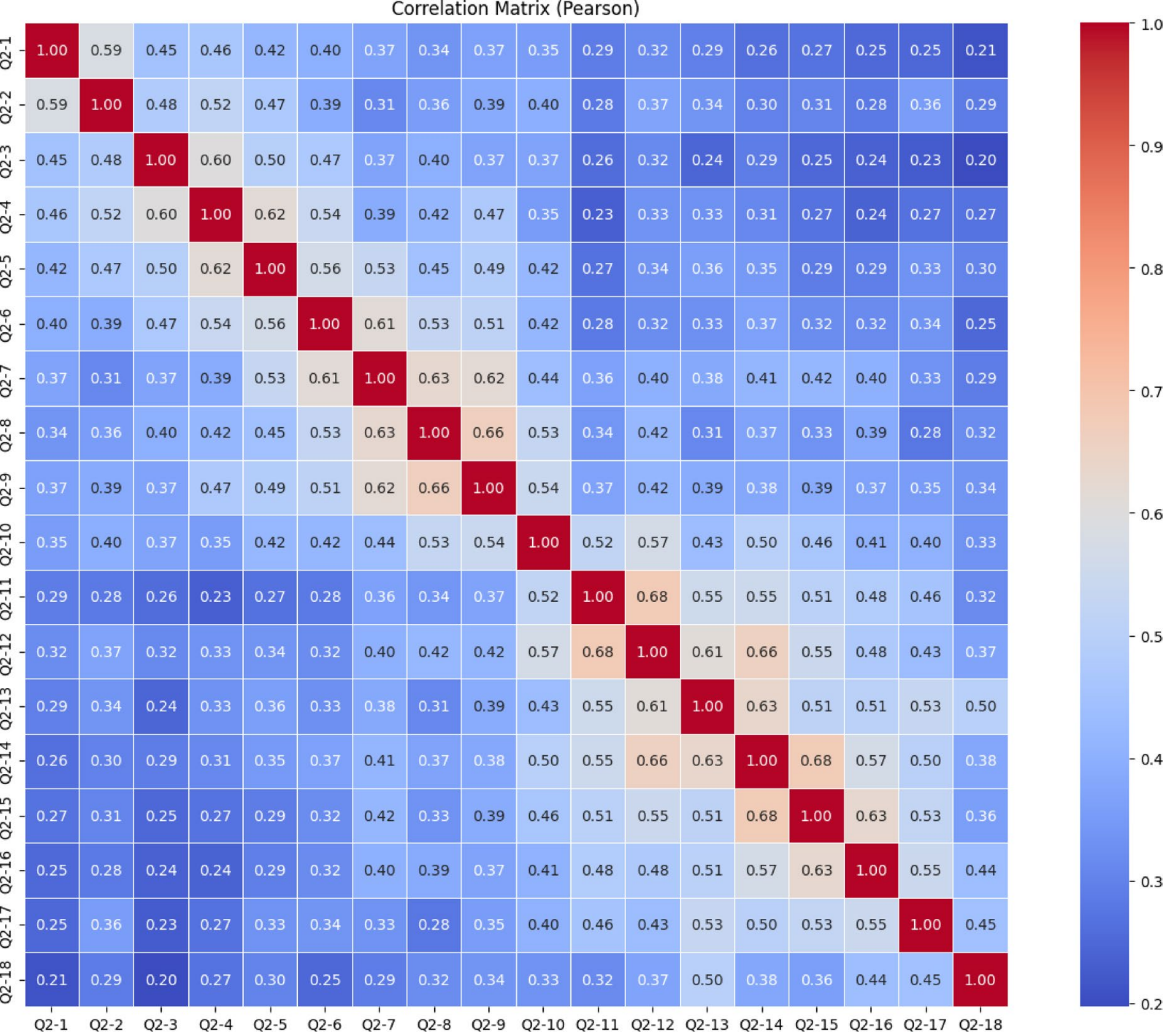
Skills sub-dimension pearson correlation heatmap.

The correlation matrix in Fig. 15 shows the Pearson correlations between items Q2-1 through Q2-18 of the Skills Subscale. Generally, moderately positive correlations (*r* ≈ 0.30–0.60) are observed between the items. This indicates that the items measure the same general skill construct and that the scale has strong structural consistency. In particular, the correlations between Q2-11 and Q2-12, Q2-12 and Q2-13, Q2-13 and Q2-14, Q2-14 and Q2-15, and Q2-15 and Q2-16 are quite high at 0.68. These items form a cluster within themselves, indicating that they reflect the same subscale. Similarly, there is a strong correlation (0.66) between Q2-8 and Q2-9. These high correlations suggest that some items can be considered together to form subscales. On the other hand, the fact that some items, such as Q2-1, Q2-2, and Q2-3, have lower correlations than others suggests that these items have more independent constructs and that the scale may be multidimensional. To statistically test the structural validity of the scale, it is recommended to conduct exploratory or confirmatory factor analysis.

###  21 st century competencies character subscale

**Fig. 16:**
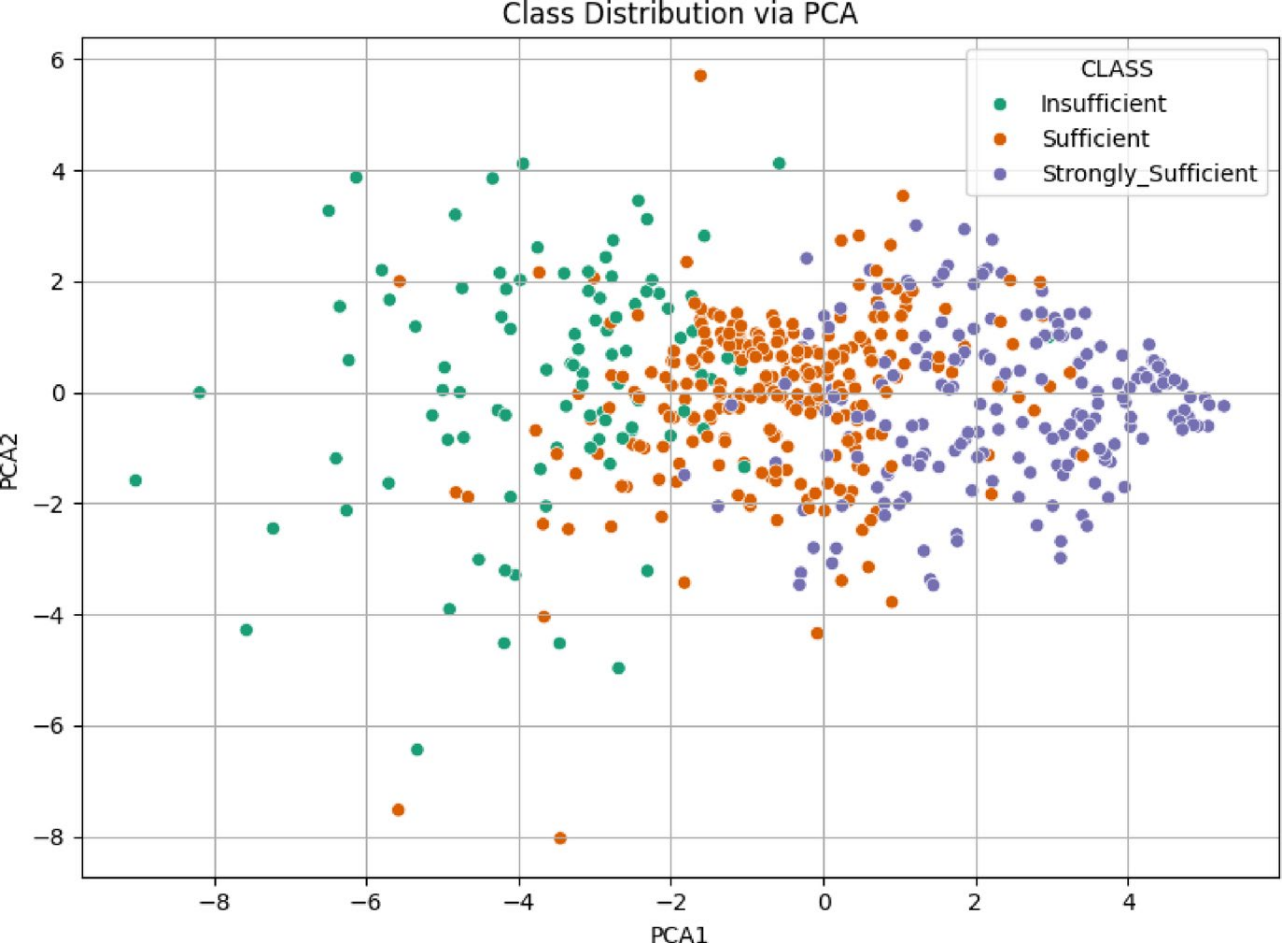
Class distribution via PCA scatter plot for character sub-dimension.

The scatter plot for the 21 st Century Competencies Character Sub-Dimension displays the class distribution based on the first two principal components obtained through PCA (Fig. 16). The Strongly Sufficient class is concentrated in the positive region of PCA1, forming a distinct cluster toward the right side of the plot (Figs. 16). In contrast, the Insufficient class is mostly located in the negative region of PCA1, though its distribution is more scattered. The Sufficient class lies between the other two, with considerable overlap on both sides. This pattern indicates a general separation trend, particularly between Insufficient and Strongly Sufficient, but also reveals areas of ambiguity due to the spread of the intermediate class.

**Fig. 17:**
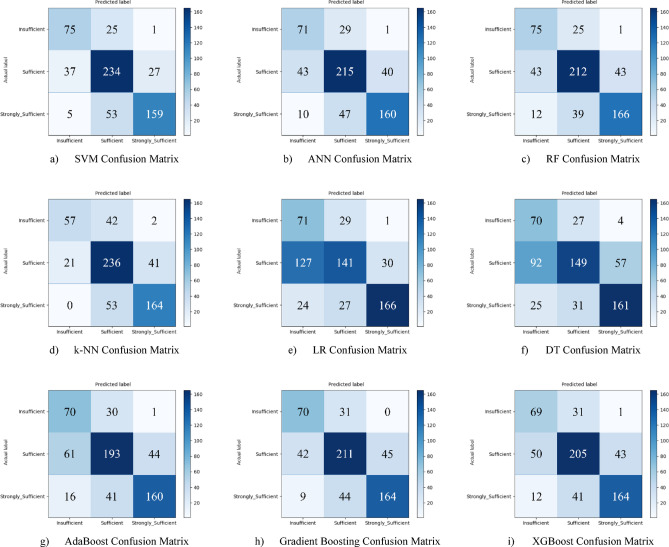
Sub-dimension (21st century competencies character subscale) confusion matrix.

In the initial analysis for the “21st Century Competencies Character Subscale,” the most successful model was SVM, with an accuracy rate of 76.0%. SVM was closely followed by k-NN (74.2%) and RF (73.5%) models, respectively. The fact that most models’ performance was concentrated in the 71–76% range indicates that generally competitive results were achieved for this subscale. As with the previous subscales, the LR and DT models produced the lowest results, around 61%. The confusion matrix is presented in the respective figures (Figs. 17(a-i)).

**Fig. 18:**
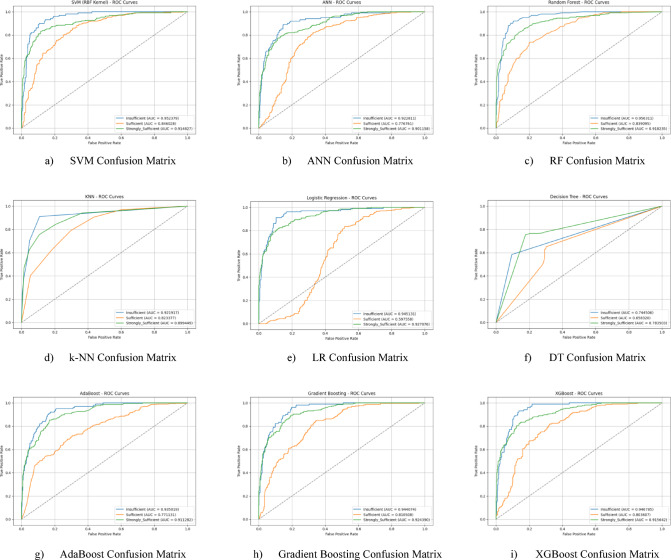
Sub-dimension (21st century competencies character sub-scale) ROC curves.

The ROC curves for all nine classification models used in evaluating the Character Sub-Dimension of 21 st Century Competencies with grid search optimization are presented in Fig. 18(a-i). These plots illustrate each model’s performance across the three class categories.

Comparative results of the models are presented in Table 6. This table clearly demonstrates that the SVM model is the most suitable model, demonstrating the highest success in all metrics. The k-NN and RF models also produced very similar and successful results.

**Table 6:** Model performance comparison table for character Sub-Scale.

Model	Accuracy (%)	Precision (%)	Recall (%)	F1-Score (%)
SVM	**75.97**	**76.75**	**75.97**	**76.13**
ANN	72.40	73.17	72.40	72.63
RF	73.54	74.46	73.54	73.77
k-NN	74.19	74.38	74.19	73.99
LR	61.36	69.55	61.36	63.02
DT	61.69	66.51	61.69	62.36
AdaBoost	68.67	70.67	68.67	69.20
Gradient Boosting	72.24	72.82	72.24	72.42
XGBoost	71.10	72.21	71.10	71.43

**Fig. 19:**
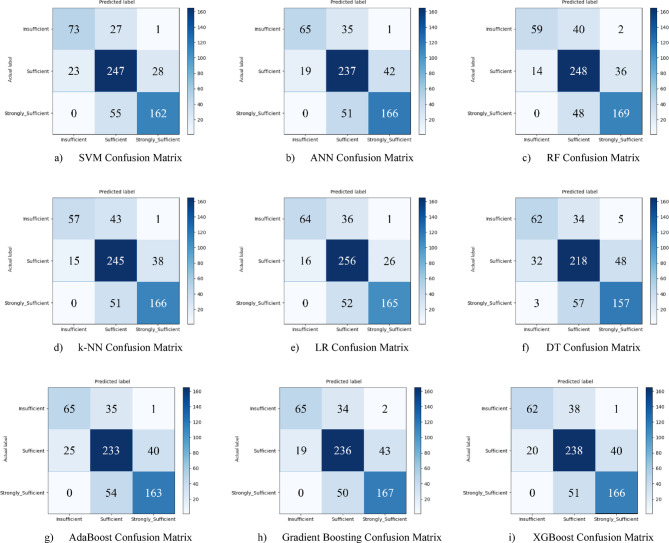
Sub-dimension (21st century competencies character subscale) grid search optimization confusion matrix.

Hyperparameter optimization using the Grid Search method took the model’s performance to the next level. The LR model achieved the highest post-optimization accuracy with 78.7%. This result is quite remarkable, demonstrating that LR, initially one of the weakest models, can become the most successful model with the right parameters. The SVM (78.2%) and RF (78.2%) models also achieved results very close to LR, proving themselves to be among the best candidates for this sub-dimension. This process improved the performance of all models, further demonstrating the potential of the dataset (Fig. 19(a-i)).

**Fig. 20:**
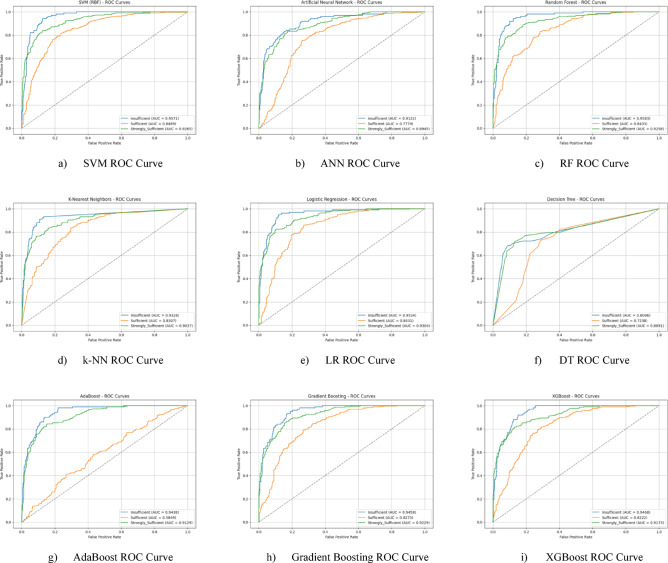
Sub-dimension (21st century competencies character sub-scale) grid search optimization ROC curves.

The ROC curves for all nine classification models used in evaluating the Character Sub-Dimension of 21 st Century Competencies with grid search optimization are presented in Fig. 20(a-i). These plots illustrate each model’s performance across the three class categories.

The results of hyperparameter optimization performed using Grid Search for each machine learning model are summarized in Table 7. This table presents the best classification accuracy obtained (%) for the Character Sub-Scale, along with the corresponding optimal parameter settings identified during the tuning process.

**Table 7:** Grid search optimization results and optimal parameters for character Sub-Scale.

Model	Best Accuracy (%)	Best Parameters
SVM	78.24	C: 1, gamma: scale, kernel: rbf
ANN	76.63	activation: relu, alpha: 0.001, hidden_layer_sizes: (50,)
RF	78.23	n_estimators: 200, max_depth: 20, min_samples_split: 5
k-NN	75.65	n_neighbors: 7, weights: distance
LR	78.73	C: 0.1, penalty: l2, solver: lbfgs
DT	71.10	max_depth: 10, min_samples_leaf: 2, min_samples_split: 5
AdaBoost	74.83	n_estimators: 50, learning_rate: 1.5
Gradient Boosting	75.97	n_estimators: 100, learning_rate: 0.1, max_depth: 3
XGBoost	75.64	n_estimators: 100, learning_rate: 0.1, max_depth: 5

**Fig. 21:**
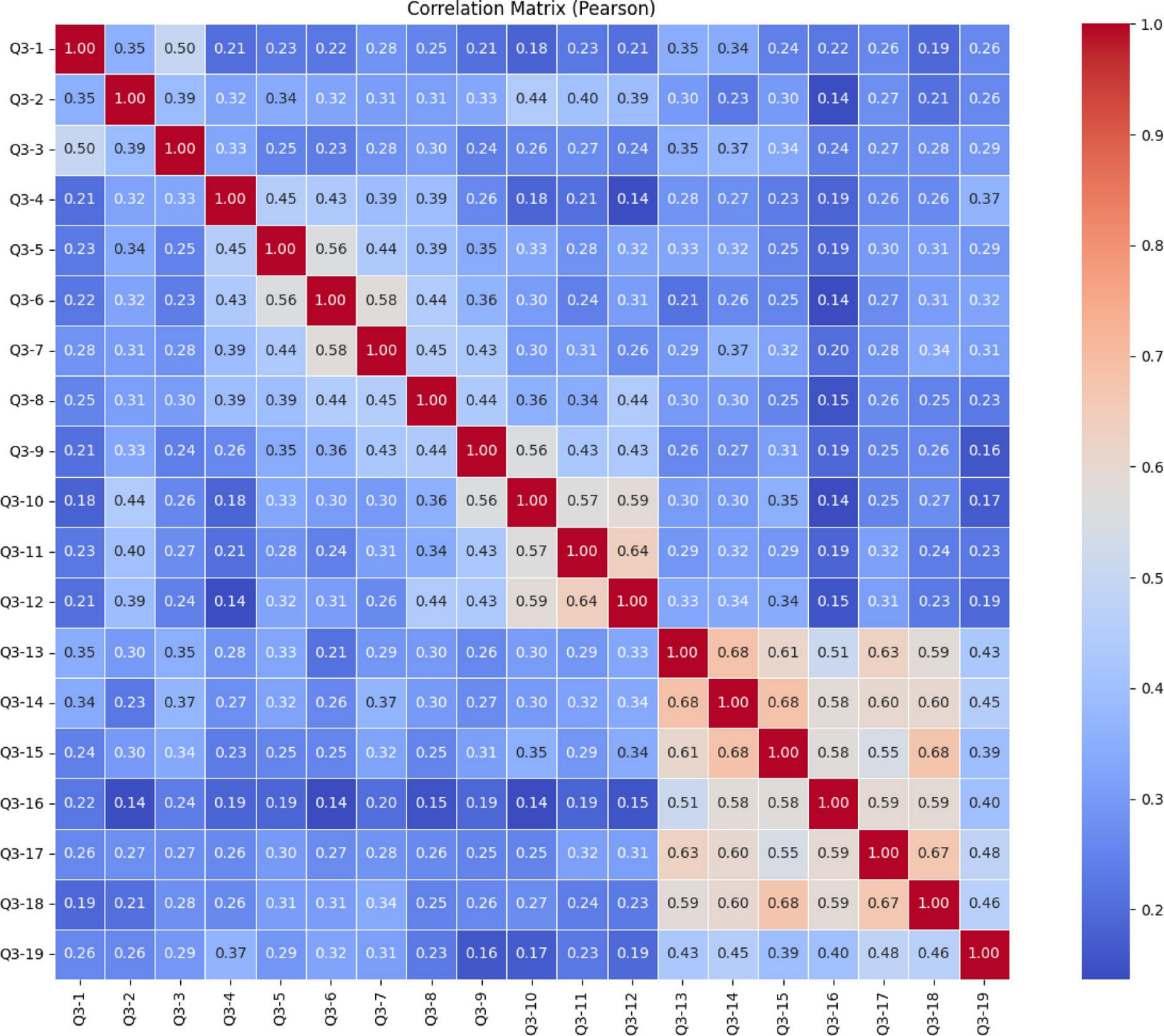
Character sub-dimension pearson correlation heatmap.

The heatmap in Fig. 21 shows the Pearson correlation between items from Q3-1 to Q3-19 of the “Character Subscale”. In general, most correlation coefficients fall between 0.20 and 0.40, indicating weak relationships between most items. This suggests that the items may be relatively independent and likely measure different aspects of the construct. However, a few moderate to strong correlations are noticeable, such as Q3-12 & Q3-13 (0.68), Q3-13 & Q3-14 (0.68), Q3-14 & Q3-15 (0.68), and Q3-17 & Q3-18 (0.67). These items likely reflect similar underlying dimensions and could potentially form subscales. Overall, there is no clear clustering among the rest of the items, which implies a multidimensional structure of the scale. To validate this structure and better understand the underlying dimensions, conducting a factor analysis is recommended.

###  21 st century competencies Meta-Learning subscale

**Fig. 22:**
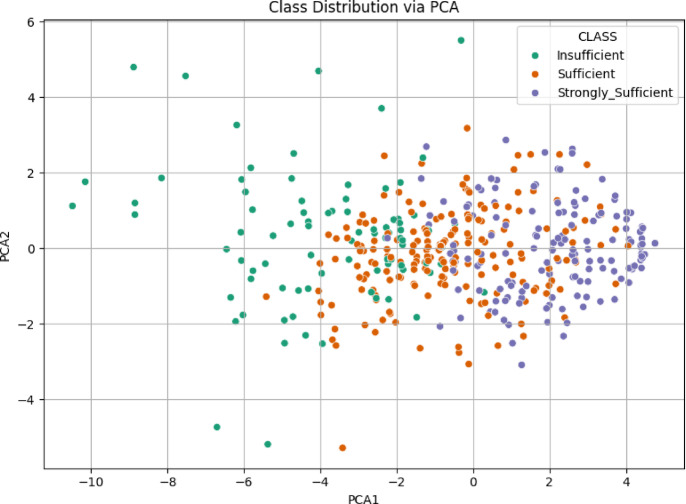
Class Distribution via PCA scatter plot for meta-learning sub-dimension.

The scatter plot for the 21 st Century Competencies Meta-Learning Sub-Dimension shown in Fig. 22, reveals the distribution of the three class categories across the first two PCA components. The Strongly Sufficient group is distinctly located on the right side of the PCA1 axis, suggesting a more coherent and concentrated structure. The Insufficient class is largely positioned on the far left, while the sufficient class is dispersed throughout the middle region, with visible overlap into both neighboring groups. This arrangement suggests a gradual transition in meta-learning proficiency across the dataset, where the boundary between classes “especially between Sufficient and the other two” is not sharply defined, and potentially complicating classification tasks.

**Fig. 23:**
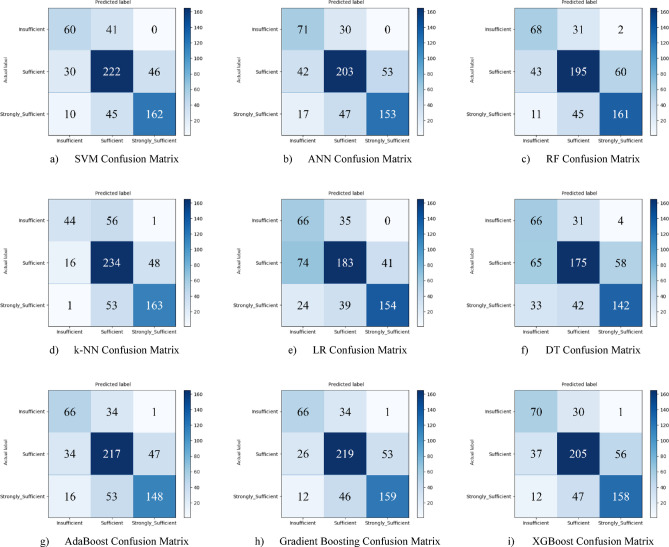
Sub-dimension (21st century competencies meta-learning subscale) confusion matrix.

In the initial modeling for the “21st Century Competencies Meta-Learning Subscale,” the SVM and Gradient Boosting models exhibited the highest initial performance with equal accuracy of 72.1%. These two models were closely followed by the k-NN model with 71.6%. The fact that most models achieved performance in the 68–72% range indicates that they achieved similar and competitive results for this subscale. As with the previous subscales, the LR and DT models produced weaker results, falling below 65%. The confusion matrix and ROC curve for each model are presented in the corresponding figures (Figs. 23(a-i)) in the text.

**Fig. 24:**
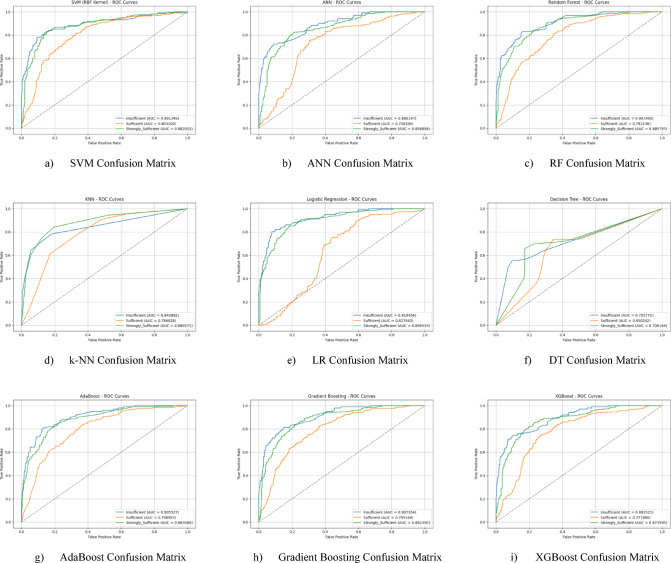
Sub-dimension (21st century competencies meta-learning sub-scale) ROC curves.

The ROC curves for all nine classification models used in evaluating the Meta-Learning Sub-Dimension of 21 st Century Competencies with grid search optimization are presented in Fig. 24(a-i). These plots illustrate each model’s performance across the three class categories.

Comparative results of the models are presented in Table 8. This table clearly demonstrates that the SVM model is the most suitable model, demonstrating the highest success across all metrics. Gradient Boosting and k-NN models also produced very similar and successful results.

**Table 8:** Model performance comparison table for meta-learning sub-scale.

Model	Accuracy (%)	Precision (%)	Recall (%)	F1-Score (%)
SVM	72.08	72.14	72.08	72.09
ANN	69.32	70.19	69.32	69.54
RF	68.83	69.38	68.83	68.93
k-NN	71.59	71.92	71.59	71.00
LR	65.42	68.87	65.42	66.40
DT	62.18	65.26	62.18	62.94
AdaBoost	69.97	69.97	69.97	69.97
Gradient Boosting	72.08	72.13	72.08	72.10
XGBoost	70.29	70.70	70.29	70.40

**Fig. 25:**
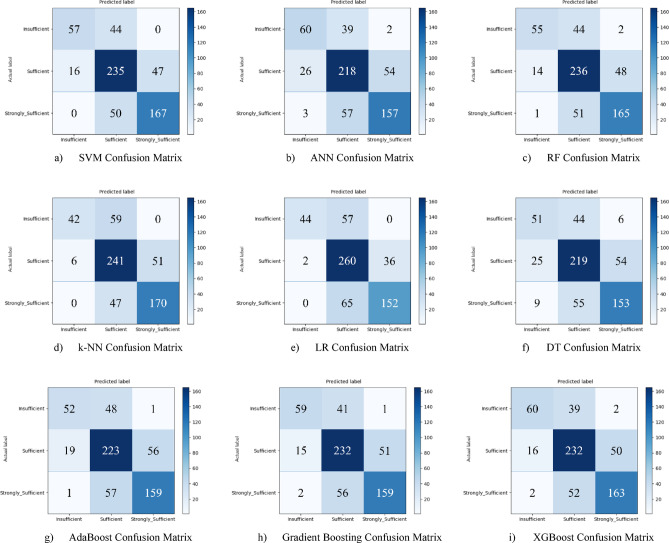
Sub-dimension (21st century competencies meta-learning subscale) grid search optimization confusion matrix.

Hyperparameter optimization using Grid Search improved the performance of all models (Fig. 25(a-i)). The SVM model achieved the highest post-optimization accuracy with 74.5%. The fact that SVM was one of the best models in this sub-dimension, both initially and after optimization, demonstrates its suitability for the data structure. The RF (74.2%) and LR (74.0%) models also achieved results very close to SVM, making them among the best candidates for this sub-dimension. The optimization process further emphasized the importance of this technique, particularly by improving the performance of the LR model by approximately 9%.

**Fig. 26:**
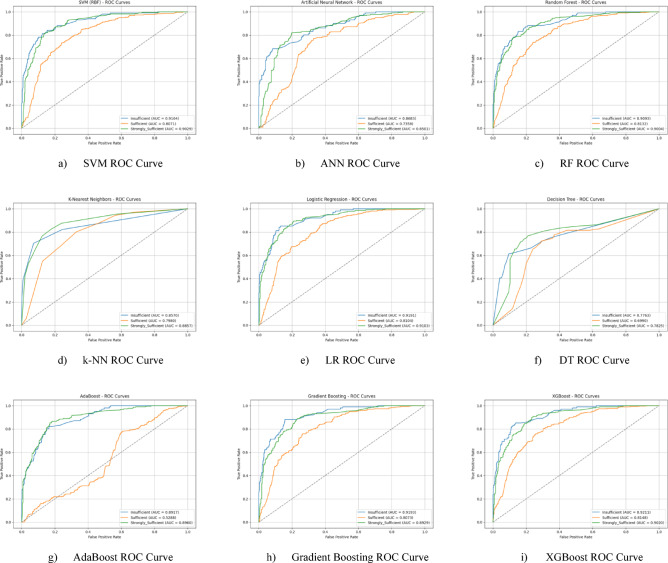
Sub-dimension (21st century competencies meta-learning sub-scale) grid search optimization ROC curves.

The ROC curves for all nine classification models used in evaluating the Meta-Learning Sub-Dimension of 21 st Century Competencies with grid search optimization are presented in Fig. 26(a-i). These plots illustrate each model’s performance across the three class categories.

The results of hyperparameter optimization performed using Grid Search for each machine learning model are summarized in Table 9. This table presents the best classification accuracy obtained (%) for the Meta-Learning Sub-Scale, along with the corresponding optimal parameter settings identified during the tuning process.

**Table 9:** Grid search optimization results and optimal parameters for Meta-Learning Sub-Scale.

Model	Best accuracy (%)	Best parameters
SVM	74.51	C: 1, gamma: scale, kernel: rbf
ANN	73.38	activation: relu, alpha: 0.0001, hidden_layer_sizes: (100, 50)
RF	74.19	n_estimators: 200, min_samples_leaf: 2, min_samples_split: 5
k-NN	73.37	n_neighbors: 7, weights: uniform
LR	74.02	C: 0.01, penalty: l2, solver: lbfgs
DT	69.32	max_depth: 10, min_samples_leaf: 1, min_samples_split: 5
AdaBoost	70.46	n_estimators: 100, learning_rate: 1.0
Gradient Boosting	73.21	n_estimators: 100, learning_rate: 0.05, max_depth: 3
XGBoost	73.86	n_estimators: 100, learning_rate: 0.1, max_depth: 3

**Fig. 27:**
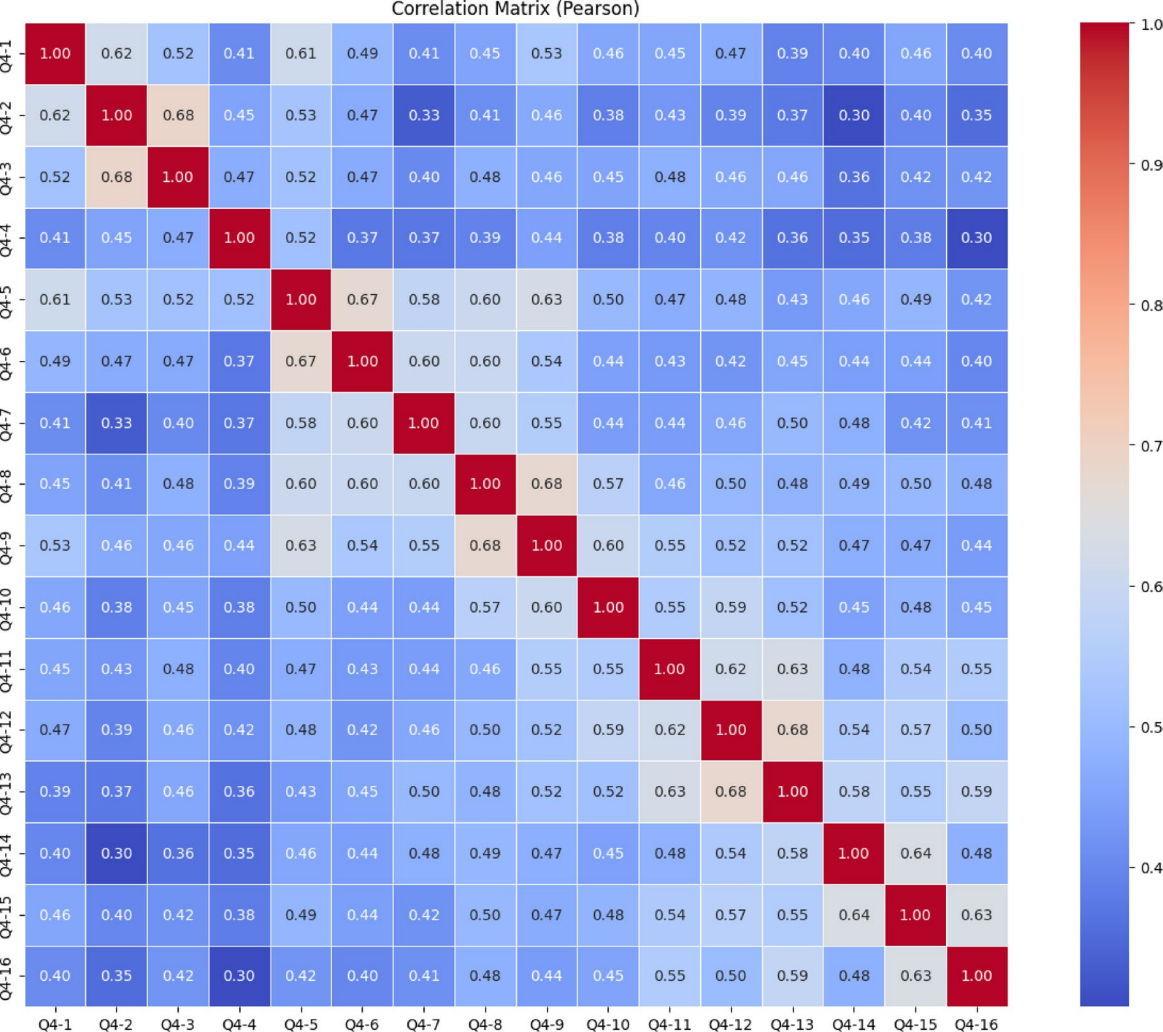
Meta-Learning Sub-Dimension Pearson Correlation Heatmap.

The heatmap in Fig. 27 illustrated the Pearson correlation matrix among 16 questionnaire items labeled Q4-1 through Q4-16. Pearson correlation values range from − 1.0 to + 1.0, indicating the strength and direction of linear relationships between items. In this matrix, all correlations are positive, suggesting a consistent direction in how participants responded across items.

Several item pairs exhibit strong positive correlations (*r* ≥ 0.60), including Q4-2 & Q4-3 (0.68), Q4-5 & Q4-6 (0.67), Q4-11 & Q4-13 (0.68), Q4-8 & Q4-9 (0.68), and Q4-14 & Q4-15 (0.64). These high correlations indicate that these items are likely measure similar or overlapping constructs and could form subscales in further analysis. However, a very high correlation between items (especially above 0.65) may suggest redundancy, which might be considered during scale refinement. Conversely, some item pairs like Q4-2 & Q4-15 (0.30), Q4-4 & Q4-16 (0.30), and Q4-2 & Q4-16 (0.35) demonstrate weak correlations, implying that they are relatively independent and may address distinct aspects of the construct being measured.

In general, the correlation values mostly fall between 0.40 and 0.60, indicating moderate relationships among most items. This balance suggests the questionnaire captures related but non-identical dimensions, which is typically desirable in psychometric tools. Based on these findings, conducting an exploratory or confirmatory factor analysis is recommended to uncover underlying constructs. Additionally, computing Cronbach’s Alpha for subsets of highly correlated items could help assess the internal consistency of the scale.

###  21 st century competencies scale

The classification models were applied both with default parameters and after hyperparameter tuning using GridSearchCV. Across all models tested (SVM, ANN, RF, LR, DT, AdaBoost, Gradient Boosting, and XGBoost), the tuned versions consistently outperformed the baseline models. Key improvements were observed in classification accuracy, F1-score, and ROC-AUC values, particularly for distinguishing the Sufficient and Strongly Sufficient classes, which had previously shown overlap in PCA distributions.

RF and Gradient Boosting models yielded the most robust performance post-tuning, effectively handling the imbalanced and overlapping class structure. LR, while interpretable, showed lower sensitivity for the Insufficient class. These results suggest that ensemble-based methods, especially with optimization, are more suitable for modeling the multidimensional structure of the 21 st Century Competencies Scale.

**Fig. 28:**
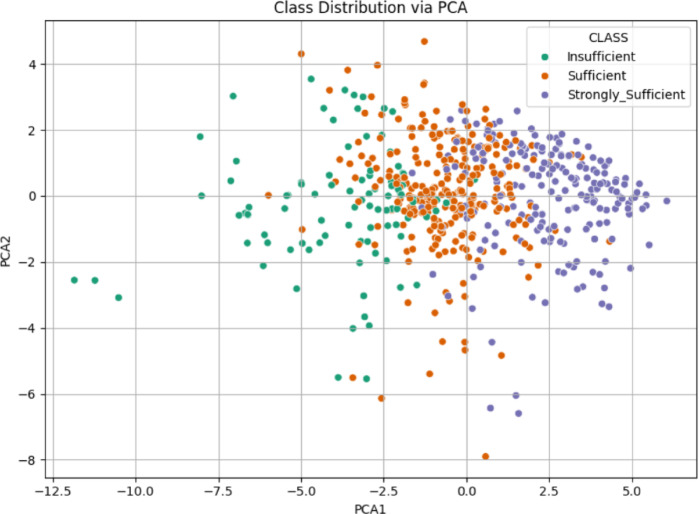
Class distribution via PCA scatter plot for competencies scale.

The PCA visualization combining all items from the four sub-dimensions in Fig. 28, illustrates a clearer pattern of class separability. The Strongly Sufficient group forms a tight cluster on the right-hand side, while the Insufficient class is more dispersed on the left. The Sufficient class remains concentrated in the center, showing moderate overlap with both neighboring categories. Compared to the individual PCA plots, this combined representation offers improved separability, especially between Strongly Sufficient and Insufficient. This finding indicates that the integration of all sub-dimensions enhances the discriminative power of the dataset and is more informative for classification purposes.

**Fig. 29:**
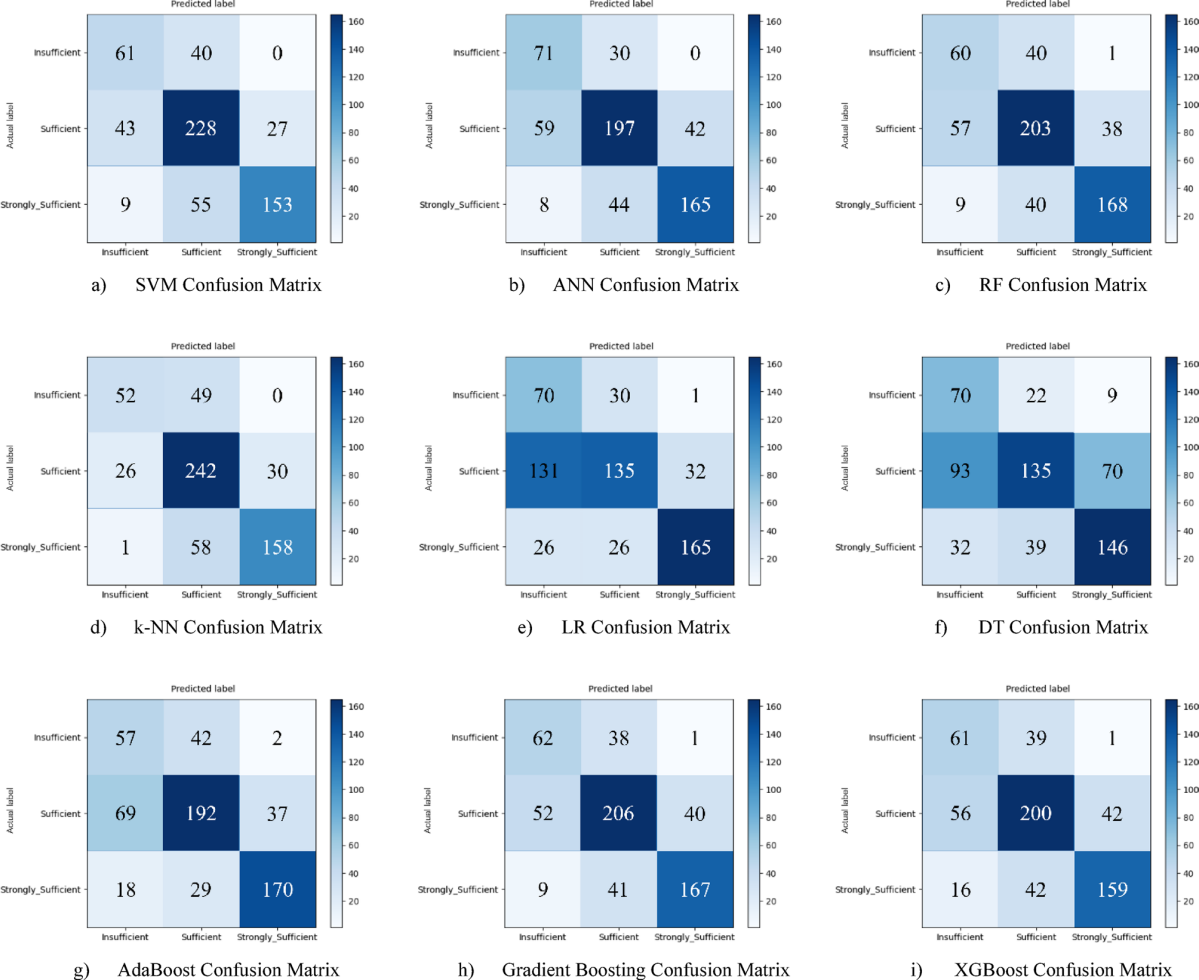
Sub-dimension (21st century competencies scales) confusion matrix.

Based on the obtained results for the combined dataset, the k-NN model is the top performer with an overall accuracy of 73.4%. The k-NN model not only achieved the highest accuracy but also a strong weighted F1-score of 0.731, showing a good balance between precision and recall across all classes. The SVM was the second-best model with an accuracy of 71.8%. It particularly excelled in correctly identifying the “strongly_sufficient” class with a high precision of 85%. Several other models delivered competitive performance, clustering around the 70% accuracy mark, including Gradient Boosting (70.6%), ANN (70.3%), and RF (70.0%). The weakest models were LR (60.1%) and, most notably, the DT (57.0%). Both models struggled significantly with precision for the “Insufficient” class, indicating they incorrectly labeled many samples into this category (Fig. 29(a-i)).

**Fig. 30:**
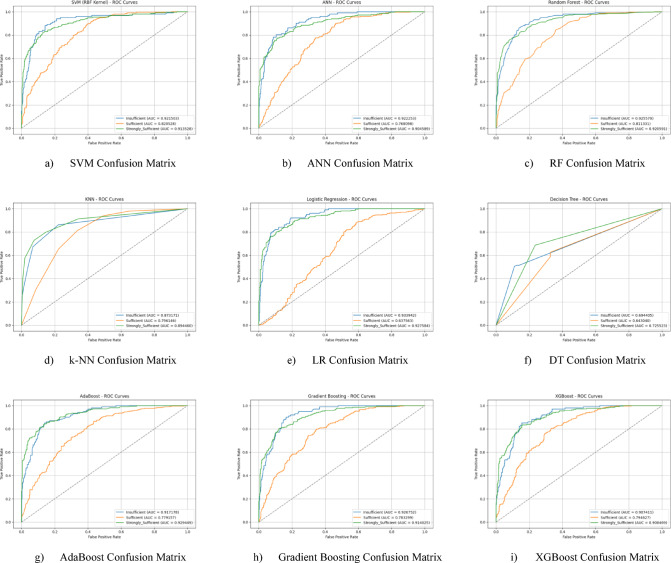
Sub-dimension (21st century competencies scales) ROC curves.

The ROC curves for all nine classification models used in evaluating all Sub-Dimension of 21 st Century Competencies with grid search optimization are presented in Fig. 30(a-i). These plots illustrate each model’s performance across the three class categories.

Comparative results of the models are presented in Table 10. This table clearly demonstrates that the k-NN model is the most suitable model, demonstrating the highest success across all metrics. The SVM and Gradient Boosting models also produced very similar and successful results.

**Table 10:** Model performance comparison table for competencies Scales.

Model	Accuracy (%)	Precision (%)	Recall (%)	F1-Score (%)
SVM	71.75	72.94	71.75	72.02
ANN	70.29	71.68	70.29	70.66
RF	69.97	71.10	69.97	70.39
k-NN	73.38	73.94	73.38	73.15
LR	60.06	68.61	60.06	61.72
DT	56.98	62.07	56.98	57.47
AdaBoost	68.02	70.46	68.02	68.86
Gradient Boosting	70.62	71.51	70.62	70.95
XGBoost	68.18	69.68	68.18	68.70

**Fig. 31:**
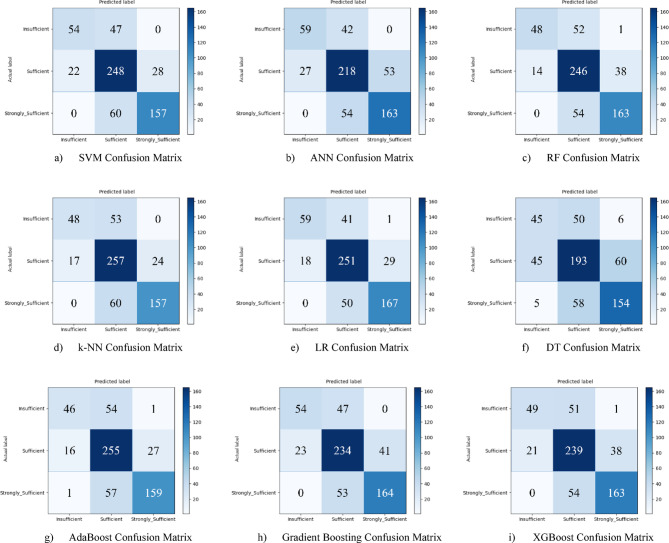
Sub-dimension (21st century competencies scale) grid search optimization confusion matrix.

Hyperparameter tuning significantly improved the performance of nearly all models compared to their baseline results. The LR model saw the most dramatic improvement, elevating it from one of the weakest performers to the best overall. A competitive group of models followed closely behind, including RF (75.3%), k-NN (75.0%), AdaBoost (74.7%), and SVM (74.5%). Their strong performance highlights that multiple model types are viable for this dataset once properly tuned. The DT model, with an accuracy of 64.3%, benefited the least from optimization and remained the weakest performer (Fig. 31(a-i)).

**Fig. 32:**
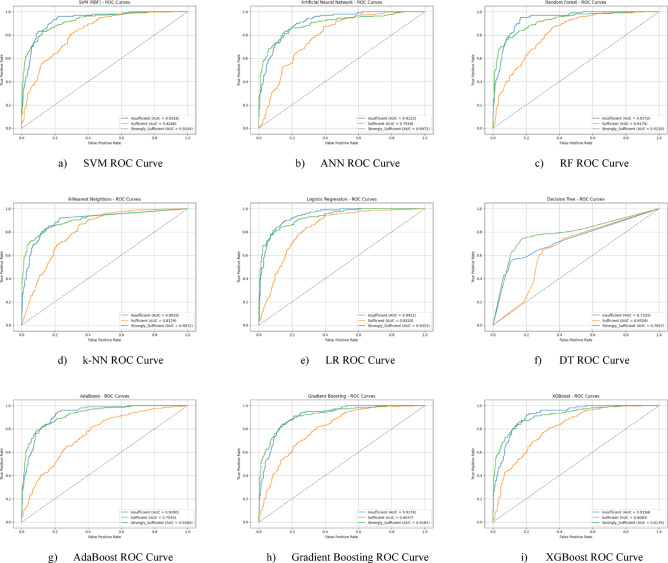
Sub-dimension (21st century competencies scale) grid search optimization roc curves.

The ROC curves for all nine classification models used in evaluating all Sub-Dimension of 21 st Century Competencies with grid search optimization are presented in Fig. 32(a-i). These plots illustrate each model’s performance across the three class categories.

The results of hyperparameter optimization performed using Grid Search for each machine learning model are summarized in Table 11. This table presents the best classification accuracy obtained (%) for the competencies all sub-scale, along with the corresponding optimal parameter settings identified during the tuning process.

**Table 11:** Grid search optimization results and optimal parameters for competencies Scale.

Model	Best Accuracy (%)	Best Parameters
SVM	74.51	C: 1, gamma: scale, kernel: rbf
ANN	73.38	activation: relu, alpha: 0.001, hidden_layer_sizes: (100, 50)
RF	75.32	n_estimators: 200, max_depth: 10, min_samples_leaf: 1
k-NN	75.01	n_neighbors: 7, weights: distance
LR	77.44	C: 0.1, penalty: l2, solver: lbfgs
DT	64.29	max_depth: 10, min_samples_leaf: 1, min_samples_split: 5
AdaBoost	74.68	n_estimators: 100, learning_rate: 0.5
Gradient Boosting	73.05	n_estimators: 100, learning_rate: 0.05, max_depth: 3
XGBoost	73.21	n_estimators: 100, learning_rate: 0.05, max_depth: 3

**Fig. 33:**
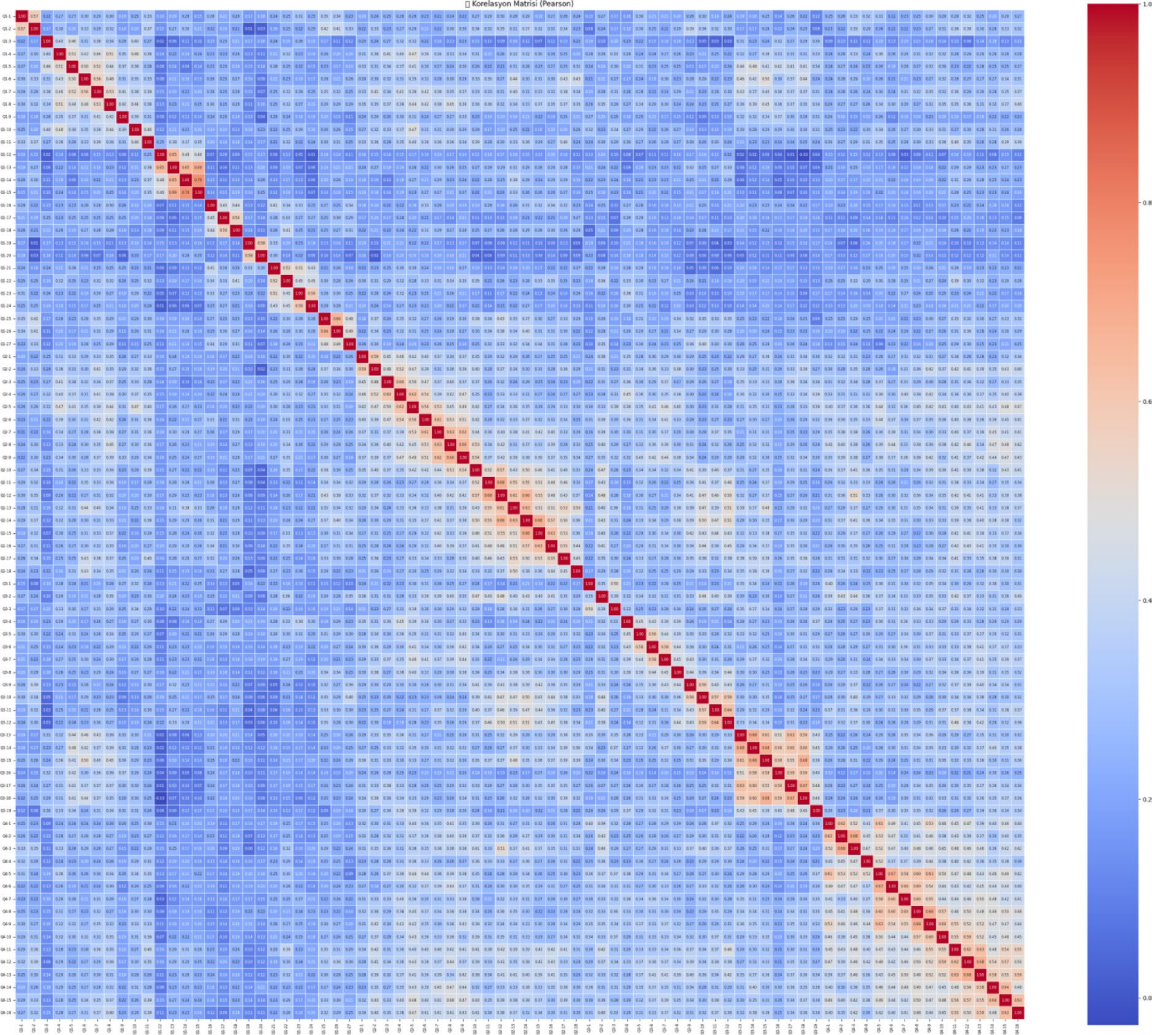
21 st century competencies scale pearson correlation heatmap.

This comprehensive Pearson correlation matrix represents the interrelationships among all items from four subscales combined into a single structure (Fig. 33). The overall pattern shows that most correlation values fall within the low to moderate range (approximately 0.20 to 0.50), indicating that while the items share some degree of association, they generally measure distinct facets of the broader construct. Certain clusters with stronger correlations are visually prominent along the diagonal, suggesting internal consistency within each subscale, particularly where items from the same group are highly interrelated. These areas of stronger correlation reflect potential sub-dimensions or thematic groupings (e.g., items from the same factor or domain). Meanwhile, the relatively weaker correlations across different subscale boundaries support the multidimensional structure of the overall instrument. This structure is desirable in multidimensional scale development, as it allows for both shared variance within domains and conceptual distinction between them. To confirm and further clarify these patterns, exploratory or confirmatory factor analysis is strongly recommended.

The Pearson correlation matrix provides a comprehensive view of the linear relationships between all items across the four sub-dimensions of the 21 st Century Competencies Scale. The matrix highlights several clusters of items with high internal consistency, as evidenced by the strong positive correlations (*r* > 0.70) along the diagonal blocks. These localized red zones indicate that related questions within the same sub-dimension are closely associated. However, correlations between items from different sub-dimensions are generally weaker (light blue), suggesting that each sub-dimension captures distinct constructs. This structure validates the conceptual division of the scale and supports its use for sub-scale-specific analysis.

**Table 12:** 21 st century competencies model accuracy Comparison.

Model	Knowledge	Knowledge Grid Search	Skills	Skills Grid Search	Character	Character Grid Search	Meta-Learning	Meta-Learning Grid Search	All Scale	All Scale Grid Search
SVM	71.75	74.51	77.92	78.58	75.97	78.24	72.08	74.51	71.75	74.51
ANN	69.81	74.36	70.62	75.00	72.40	76.63	69.32	73.38	70.29	73.38
RF	71.59	75.49	74.84	77.44	73.54	78.23	68.83	74.19	69.97	75.32
k-NN	73.38	75.01	75.16	75.49	74.19	75.65	71.59	73.37	73.38	75.01
LR	60.06	77.44	68.67	78.58	61.36	78.73	65.42	74.02	60.06	77.44
DT	56.49	65.10	65.75	72.4	61.69	71.1	62.18	69.32	56.98	64.29
AdaBoost	68.02	74.68	70.29	75.49	68.67	74.83	69.97	70.46	68.02	74.68
Gradient Boosting	70.94	73.21	76.30	76.95	72.24	75.97	72.08	73.21	70.62	73.05
XGBoost	68.18	73.21	71.11	76.95	71.10	75.64	70.29	73.86	68.18	73.21

**Fig. 34:**
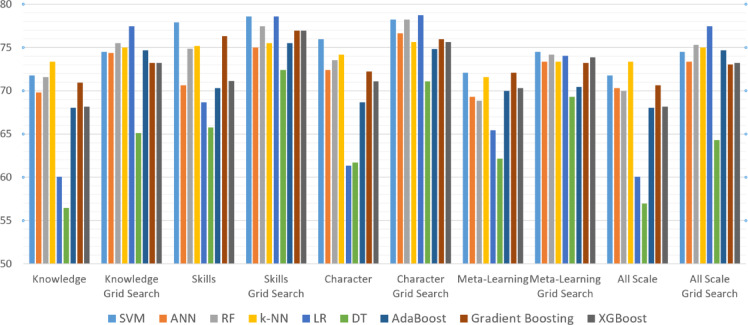
21 st century competencies model accuracy comparison- bar chart.

Table 12 presents a comparison of classification accuracy results for nine different machine learning models across various sub-dimensions of the 21 st Century Competencies Scale, both with and without grid search optimization. Overall, the results show that applying grid search consistently improves model performance across all sub-dimensions. Among the models, SVM and Gradient Boosting achieved the highest accuracy in most dimensions after optimization, showing strong ability. k-NN also performed well, especially in the non-optimized runs, suggesting its effectiveness with the dataset structure. Logistic Regression, while initially showing lower accuracy values, gained significant improvements with grid search, even outperforming several other models in the optimized setting. Decision Tree, on the other hand, showed the lowest performance overall, though it also benefited from parameter tuning. The ANN, Random Forest, and XGBoost models maintained balanced performance throughout, with moderate to high accuracy rates. These results suggest that model selection and parameter tuning both play a critical role in classification performance, and SVM, Gradient Boosting, and optimized Logistic Regression models appear to be the most promising approaches for this specific task. Figure 34 presents the 21 st Century Competencies Model Accuracy Comparison – Bar Chart, which illustrates the comparative accuracy levels of the models.

Across all analyses, the comparative evaluation revealed consistent patterns regarding model performance. Ensemble-based methods (Random Forest, Gradient Boosting, XGBoost) and kernel-based algorithms (SVM) generally demonstrated robust and stable accuracy across the four sub-dimensions as well as the combined dataset. The k-NN model also achieved strong baseline results, particularly in the Knowledge and Skills subscales. Logistic Regression, although initially one of the weaker performers, improved significantly after hyperparameter tuning, achieving the highest accuracy (78.7%) in the Character subscale. This suggests that students’ self-reported dispositions, such as responsibility, empathy, and resilience, may align in a relatively linear fashion with the survey items, allowing a simpler model to capture these patterns effectively. Conversely, Decision Tree models yielded the lowest performance across most settings, even after optimization. Interestingly, XGBoost underperformed in certain sub-dimensions compared to Random Forest and Gradient Boosting. This discrepancy may be attributed to the relatively small sample size and the ordinal nature of Likert data, as boosting methods are more sensitive to noise and sparsity, making them less effective in capturing stable patterns across all subscales. Collectively, these findings suggest that optimized ensemble and kernel-based models are better suited for modeling multidimensional competency scales, while simpler algorithms may require careful parameterization to achieve competitive results.

Beyond the numerical performance of the models, the results carry important implications for competency-based education. The relatively high classification accuracy in the Knowledge and Skills sub-dimensions suggests that these domains are well captured by structured survey responses and can be effectively modeled by classical machine learning algorithms. This indicates that educators may reliably use such instruments to identify students who require additional support in academic content mastery and applied problem-solving. In contrast, the comparatively lower performance in the Character and Meta-learning dimensions reflects the inherent complexity of assessing personal dispositions, ethical values, and self-regulation skills. These competencies may not be fully observable through Likert-type self-report measures and could require complementary assessment methods (e.g., behavioral learning analytics, longitudinal evaluations, or teacher assessments). Importantly, the findings suggest that predictive modeling can serve as a decision-support tool rather than a replacement for human judgment. By highlighting areas where students are classified as “Insufficient,” instructors and academic advisors can target interventions more effectively. For example, a student flagged as insufficient in Meta-learning may benefit from tailored coaching on study strategies, time management, or reflective learning practices.

## Conclusions

This study systematically evaluated nine machine learning models (SVM, ANN, RF, k-NN, LR, DT, AdaBoost, Gradient Boosting, and XGBoost) for classifying students’ competencies using the 21 st Century Competencies Scale. The findings showed that model performance varied considerably across algorithms and sub-dimensions, underscoring the importance of both algorithm choice and hyperparameter optimization. For example, the k-NN model achieved the highest baseline accuracy in the Knowledge subscale (73.4%), while the SVM model was most effective in the Skills subscale (77.9%). Logistic Regression, initially among the weakest models, achieved the highest overall performance in the Character subscale (78.7%) after optimization. In the Meta-learning subscale, SVM and Gradient Boosting produced the strongest results (74.5% after optimization). For the combined dataset, optimized Logistic Regression improved from a baseline accuracy of 60.1% to 77.4%, highlighting the importance of parameter tuning.

The study makes three main contributions. First, it provides a comprehensive comparative framework for evaluating classical machine learning algorithms on multidimensional educational datasets. Second, it demonstrates that ensemble and kernel-based approaches, particularly Random Forest, Gradient Boosting, and SVM, consistently yield robust performance across subscales. Third, it shows that hyperparameter optimization can substantially improve even relatively simple models, such as Logistic Regression, allowing them to compete with more complex algorithms.

Despite these contributions, several limitations must be acknowledged. The dataset was derived exclusively from self-report Likert-type responses, which may not fully reflect behavioral or contextual indicators of competencies. The voluntary nature of participation introduces potential selection bias, limiting the strength of findings. Furthermore, the study was restricted to classical machine learning methods and did not examine deep learning or explainable AI approaches.

Future research should build upon this work by integrating behavioral learning traces (e.g., LMS logs, interaction data), validating results with more diverse and stratified student populations, and incorporating explainable AI methods to enhance both interpretability and pedagogical applicability. The results of this study demonstrate the promise of AI-driven data mining approaches for competency-based assessment, provided that they are applied with methodological rigor and transparent reporting.

The findings of this study extend beyond methodological contributions and offer practical implications for higher education. First, the demonstrated ability of machine learning models to classify students into *Insufficient*,* Sufficient*, and *Strongly Sufficient* categories provides educators with an evidence-based approach to identify students at risk of underperforming in key 21 st -century competencies. This can guide targeted support interventions, such as remedial courses, mentoring, or skill-development workshops.

Second, the comparative performance of models across sub-dimensions informs curriculum design. For example, the stronger predictability of *Knowledge* and *Skills* highlights areas where standard instruction and assessments are effective, while the more challenging domains of *Character* and *Meta-learning* suggest the need for innovative pedagogical strategies, such as experiential learning, project-based activities, and reflective practices.

Third, at the policy level, this study demonstrates the potential of AI-driven analytics to complement large-scale educational assessments. By incorporating predictive modeling into institutional dashboards, policymakers can monitor competency development trends and allocate resources more efficiently. However, such applications must be balanced with ethical safeguards, ensuring fairness, transparency, and the responsible use of student data.

Methodological limitations of this study should also be acknowledged. First, the dataset is entirely based on self-reported Likert-type questionnaire responses, which means that the observed patterns primarily reflect students’ perceptions rather than their actual behavioral traces. While this provides structured and standardized measures, it limits the scope of inference and reduces the direct applicability of the findings in real-time learning environments. Second, the sample consists of 616 students from various universities in Turkey. Although this number is substantial, it represents a single national context, which may affect the generalizability of the findings to other cultural and institutional settings. Third, although multiple machine learning algorithms were optimized and evaluated, the results are dependent on the chosen hyperparameters and the specific 80-item instrument, which may limit broader applicability. These limitations imply that predictive models should be used as decision-support tools rather than replacements for human judgment.

Future research should address the identified limitations by incorporating behavioral learning data (e.g., LMS logs, interaction traces, teacher evaluations), expanding to larger and more diverse populations, and validating results with alternative instruments to ensure robustness across contexts. Beyond these methodological improvements, realistic applications should also be pursued. One promising avenue is the integration of predictive models into LMS platforms, where outputs can automatically flag at-risk students and trigger early interventions. Another is the development of adaptive dashboards that allow educators to monitor competencies across sub-dimensions in real time and allocate support more efficiently. Personalized feedback systems could further transform model outputs into actionable guidance, offering students tailored recommendations such as study strategies, mentoring, or workshops. When combined with explainable AI (XAI) methods, these applications would not only enhance predictive accuracy but also improve transparency and trust among educators and learners. Finally, longitudinal studies and cross-cultural validations of the 21 st Century Competencies Scale would strengthen the evidence base and adapt the findings to international educational contexts.

## Data Availability

The data used to support the findings of this study are available on the https://nigmetkoklu.com/datasets/21st_Century_Competency_Dataset.zip
